# Phylogenomics reveals the evolution, biogeography, and diversification history of voles in the Hengduan Mountains

**DOI:** 10.1038/s42003-022-04108-y

**Published:** 2022-10-25

**Authors:** XiaoYun Wang, Dan Liang, XuMing Wang, MingKun Tang, Yang Liu, ShaoYing Liu, Peng Zhang

**Affiliations:** 1 State Key Laboratory of Biocontrol, School of Life Sciences, Sun Yat-Sen University, Guangzhou, China; 2 Sichuan Academy of Forestry, Chengdu, China; 3 Southern Marine Science and Engineering Guangdong Laboratory (Zhuhai), Zhuhai, Guangdong Province China

**Keywords:** Phylogenetics, Zoology

## Abstract

The Hengduan Mountains (HDM) of China are a biodiversity hotspot whose temperate flora and fauna are among the world’s richest. However, the origin and evolution of biodiversity in the HDM remain poorly understood, especially in mammals. Given that the HDM shows the highest richness of vole species in the world, we used whole-exome capture sequencing data from the currently most comprehensive sampling of HDM voles to investigate their evolutionary history and diversification patterns. We reconstructed a robust phylogeny and re-estimated divergence times of the HDM voles. We found that all HDM voles could be divided into a western lineage (*Volemys*, *Proedromys*, and *Neodon*) and an eastern lineage (*Caryomys* and *Eothenomys*), and the two lineages originated from two migration events from North Eurasia to the HDM approximately 9 Mya. Both vole lineages underwent a significant acceleration of net diversification from 8–5 Mya, which was temporally congruent with the orogeny of the HDM region. We also identified strong intertribal gene flow among the HDM voles and hypothesized that frequent gene flow might have facilitated the speciation burst of the HDM voles. Our study highlights the importance of both environmental and biotic factors in shaping the biodiversity of mammals in mountain ecosystems.

## Abstract (web summary)

Phylogenomic analysis of voles in the Hengduan Mountains of China reveal two distinct genetic lineages emerging from distinct Eurasian migration events.

## Introduction

**Fig. 1:**
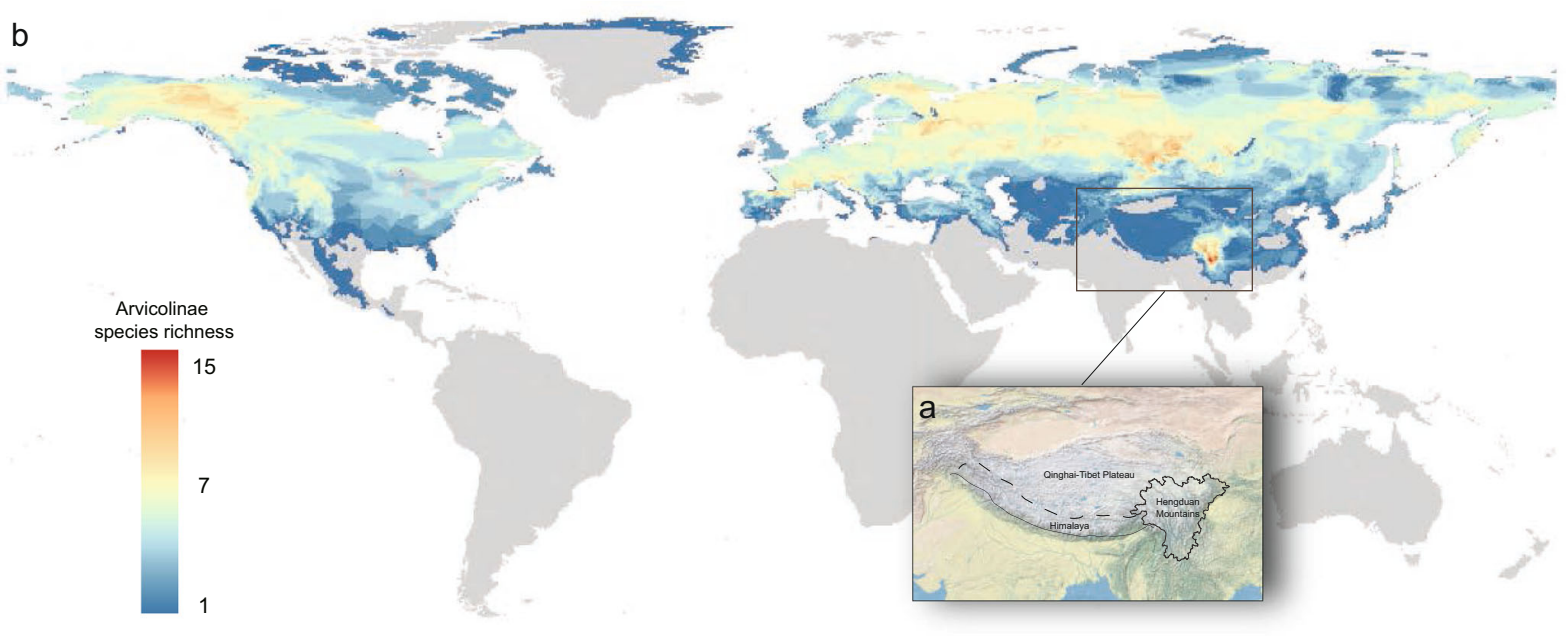
a Geographic location of the Hengduan Mountains. b Global species richness map of Arvicolinae. Grid cells (50 × 50 km) in red show the highest richness, whereas blue indicates the lowest richness. The distribution data of global Arvicolinae species were obtained from the literature^21^, online databases (IUCN red list of threatened species) and our field work record.

Despite covering only approximately one–eighth of the Earth’s land surface, mountain regions harbor one–third of all global terrestrial species^1,2^. Why do so many species occur in mountains? A common hypothesis is that mountain uplift drives the rapid speciation of organisms because orogeny creates a complex range of topographies, climates and habitats where species evolve and diversify^3–7^. On the other hand, biological factors such as biome changes associated with orogeny and genetic admixture among lineages may also contribute to the speciation process^8,9^. Illuminating the contribution of both environmental and biological factors during the development of the remarkable biodiversity in mountains is important for understanding how evolutionary processes interact with changing global environments to shape biodiversity. Among the many mountain ecosystems, the Hengduan Mountains (HDM) are an unusual, enigmatic biodiversity hotspot located in the southeastern corner of the Qinghai–Tibetan Plateau (QTP)^6,10,11^ (Fig. 1a). The temperate flora and fauna of the HDM region are among the world’s richest, including approximately 12,000 species of vascular plants and 1,500 terrestrial vertebrates, many of which are endemic^10^. Elucidating the evolutionary mechanisms driving the formation of biodiversity in the HDM has long attracted the attention of evolutionary biologists. Central to addressing this question is knowledge of the speciation tempo (rate) and mode (colonization via dispersal or in situ lineage diversification) of the resident lineages of the HDM^6^. To this end, scientists have performed a great deal of work on plants^6,7,12^, amphibians^13^, and birds^14–16^ to determine the relationship between mountain uplift and species diversification in the HDM region. In contrast, although mammals are the flagship group of terrestrial vertebrates, mammals in the HDM region have been the subject of relatively few investigations addressing their diversification tempo and mode.

Arvicolinae (Rodentia: Cricetidae) is a subfamily of rodents that includes voles, lemmings, and muskrats. It is a highly diverse, young, fast-evolving rodent group comprising ten tribes, 28 genera and over 150 species^17,18^, with new species constantly being discovered and described^19–21^. Arvicolinae are widespread in various landscapes of the Northern Hemisphere but are mainly concentrated in mountain areas and show several species diversity hotspots, including the North Rocky Mountains, the Mountains of Central Asia, and the HDM (Fig. 1b). Remarkably, the HDM region harbors the world’s richest species diversity of Arvicolinae, including ~35 species of voles, most of which are endemic. Intriguingly, the HDM region exhibits a unique island-like pattern of vole species richness, in which adjacent areas show extremely low species numbers, while the other two species diversity hotspots show gradual declines in species richness toward their adjacent areas (Fig. 1b). The unusual distribution pattern and high richness of vole species in the HDM provide an ideal mammalian model system for exploring the processes that gave rise to the biodiversity of the HDM. When and how did vole species accumulate in the HDM region? To answer this question, a robust phylogenetic framework and evolutionary timescale of Arvicolinae that includes all HDM vole species, is essential.

Although the phylogeny and evolutionary timescale of Arvicolinae have been studied for decades using both morphological and molecular data, there are important issues that remain to be addressed. First, from the perspective of large-scale systematic frameworks of Arvicolinae, the interrelationships among tribes remain unresolved. The poor resolution of many nodes in the Arvicolinae phylogeny is likely a result of the small number of molecular markers used and/or high rates of missing data in these analyses (e.g., eight genes, 9,002 bp with 72.8% missing data or 11 genes, 15,535 bp with 75% missing data)^22,23^. Whole mitochondrial genomes with low missing data rates have also been applied^24^, but mitochondrial genes are genetically linked and highly compositionally heterogenous. Second, a general consensus on the divergence times of the major Arvicolinae lineages is also lacking. The known fossil records suggest that extant arvicolids presumably emerged in the late Miocene—early Pliocene (~8–5 Mya). However, molecular data have produced a wide range of estimated origin times of extant Arvicolinae, from older estimates of ~15.2–20.9 Mya^23,25–27^ to much younger estimates of ~7 Mya^24,28^. Third, due to the difficulty of sample collection, previous studies on the phylogenetic relationships and divergence times of Arvicolinae have typically included only limited taxon sampling of HDM vole species. All of the above issues have prevented the reconstruction of a reliable, comprehensive evolutionary history of HDM vole species, which is crucial for understanding the macroevolutionary and ecological processes that shape their diversity in the HDM region.

The aims of this study were to reconstruct the evolutionary history and diversification process of HDM voles and to discuss the relationships among species biodiversity, genetic exchange, and mountain uplift in the HDM. To do this, we collected 121 rodent specimens including all known HDM vole species and used a whole-exome sequencing technique to generate genome-scale DNA sequence data. The phylogenetic analysis of these data led to a well-supported hypothesis of the relationships among voles that was highly concordant across multiple analytical approaches. With this phylogenetic framework, we further investigated the divergence times, biogeographic history, tempo and mode of diversification, and gene flow of the HDM voles to reveal the environmental and biotic factors that have driven their evolution and diversification.

## Results and discussion

### Data processing and the datasets

Based on whole-exome capture sequencing, we obtained a total of 391.7 GB of Illumina PE150 sequencing data for 115 Arvicolinae and 6 Cricetinae samples, with an average of 3.2 GB of data per sample. For each sample, ~8.84% of read sequences could be mapped to the reference coding sequences (CDSs). The number of extracted CDSs ranged from 915 (*Alticola argentatus* 26051) to 18,997 (*Myopus schisticolor* 09RAP055), with an average of 10,958 CDSs per sample (Supplementary Fig. S1). At the species level, we obtained at least 6000 CDSs per species, except for the species *Alticola argentatus*. In addition, we extracted 117 mitochondrial genomes (four samples failed) from our capture data. The extracted CDSs for each sample were deposited in the Mendeley Data Repository (https://data.mendeley.com/datasets/mwyj4m963h), and the newly sequenced mitochondrial genomes were deposited in GenBank (for accession numbers, see Supplementary Table S1). These CDS and mitochondrial genome data will be a useful and convenient resource for future studies on the biology, classification, and adaptive evolution of Arvicolinae.

We constructed three datasets from these CDS and mitochondrial genome data. The individual-level nuclear dataset contained 6517 CDSs generated entirely within this study, comprising sequences from 115 Arvicolinae and 7 Cricetinae individuals (12,041,474 nt in length and 48.2% complete), which were used to provide a basic framework for the phylogeny of Arvicolinae, emphasizing the voles in the HDM. The species-level nuclear dataset was obtained by merging the data of all individuals from each nominal species and included 6078 CDSs from 58 Arvicolinae and three Cricetinae species (10,788,858 nt in length and 69.6% complete), with the aim of reducing missing data. Then, the mitochondrial genome dataset contained the 117 mtDNA sequences newly generated in this study and 98 published mtDNA sequences, comprising sequences from 197 Arvicolinae taxa and 18 Cricetinae outgroup taxa (15,160 nt in length and 97.9% complete). It included more tribes than the nuclear datasets and can provide a more comprehensive framework for the phylogeny of Arvicolinae.

### Phylogeny of arvicolids, with emphasis on voles in the HDM

**Fig. 2:**
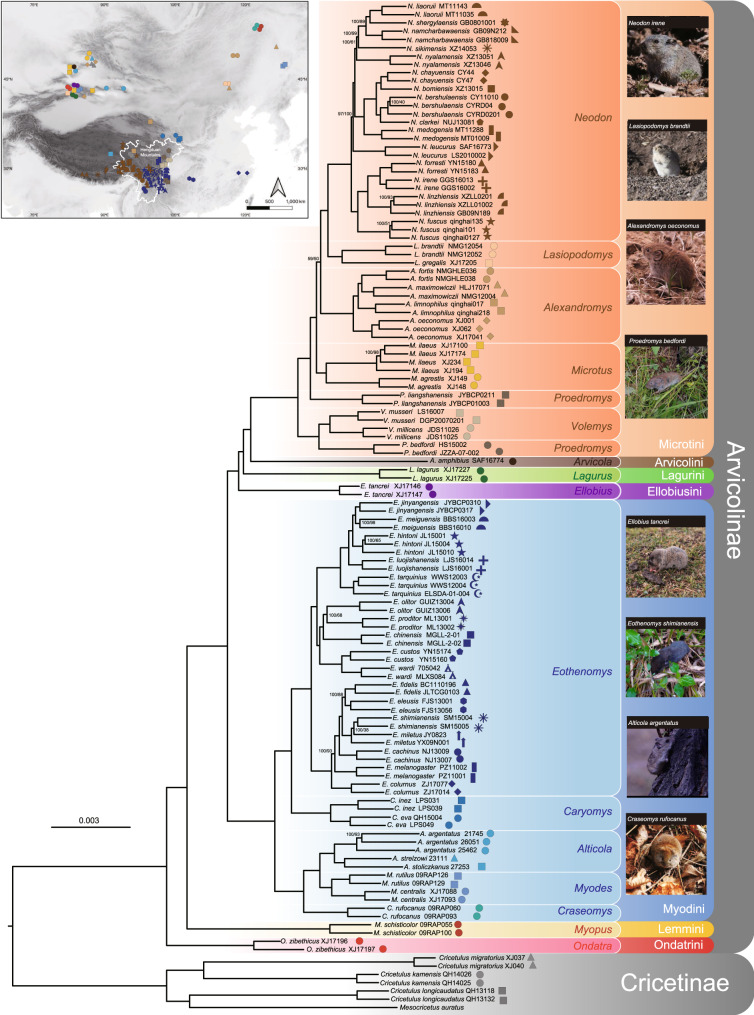
The phylogeny of Arvicolinae with a focus on the HDM taxa. The phylogenetic tree was inferred from the individual-level nuclear dataset (6517 genes, 122 taxa) through maximum likelihood analysis in IQ-TREE, which is identical to that estimated by ASTRAL. Cricetinae is used as outgroup. The branch supports are provided beside the nodes, indicating ultrafast bootstrap (UFBS) values from IQ-TREE (left) and standard bootstrap values from ASTRAL (right). The nodes without support have UFBS values and standard bootstrap values both equal to 100%. The collection localities of the vole specimens used in this study are shown at the top left (the detailed collection map is given in Fig. S9). Photo credit: *Neodon irene*, *Lasiopodomys brandtii*, *Ellobius tancrei* (Shaoying Liu); *Alexandromys oeconomus* (Yahui Huang); *Proedromys bedfordi*, *Eothenomys shimianensis* (Yingxun Liu); *Alticola argentatus* (Yang Liu); *Craseomys rufocanus* (Dingqian Xiang).

The concatenated maximum likelihood (IQ-TREE) analysis of the individual-level nuclear dataset produced a well-resolved arvicolid phylogeny, with 117 of the 119 nodes showing UFBS values = 100% (Fig. 2). The species tree analysis (ASTRAL) of this dataset produced an identical phylogeny, with 87.4% of nodes showing bootstrap values = 100% (Fig. 2). The phylogenetic trees of the species-level nuclear dataset were completely congruent with those of the individual-level nuclear dataset and showed a higher resolution: 92% of nodes had UFBS values = 100% and ASTRAL bootstrap values = 100% (Supplementary Fig. S2). To investigate whether the branches with high nodal support were robust, we estimated the gene concordance factors (gCFs) and site concordance factors (sCFs) of each branch of both the individual and species-level phylogenies. The spatial correlations between the gCF, sCF and bootstrap values showed that high bootstrap values always coincided with high gCF and sCF values, which suggested that the phylogenetic signals of the two nuclear datasets were strong and congruent and that the resulting phylogenies were robust (Supplementary Fig. S3 and Supplementary Table S2). Finally, the ML tree inferred from the mitochondrial genome dataset was essentially congruent with the results of the nuclear datasets, but the support for the deep nodes (among tribes) was not strong (UFBS < 95%) (Supplementary Fig. S4).

The placement of the genus *Arvicola* is one of the most controversial issues concerning the phylogenetic relationships of Arvicolinae. Traditionally, *Arvicola* has been considered a member of tribe Arvicolini^18^. Molecular studies based on a few mitochondrial and nuclear genes either supported^29–31^ or opposed^28,32^ this classification, but none of them received strong support. Based on mitochondrial genomes, Abramson et al. ^24^. found that *Arvicola* clustered with Lagurini, but they considered this result to be a phylogenetic artifact related to nucleotide composition bias or a long-branch attraction effect. Our mitochondrial genome tree did not robustly resolve the placement of *Arvicola* (Supplementary Fig. S4), implying insufficient mtDNA information to answer this question. In comparison, our nuclear tree based on thousands of nuclear genes strongly recovered *Arvicola* as the sister group of all other members of Arvicolini (Fig. 2), supporting the traditional classification. However, because the branch separating *Arvicola* and other members of Arvicolini was rather long and the genetic distances from *Arvicola*, Lagurini and Ellobiusini to the other members of Arvicolini were similar (Fig. 2), we suggested that the traditional Arvicolini be split into two tribes (Arvicolini and Microtini), following the suggestion of Liu et al. ^32,33^. The type genera of Arvicolini and Microtini should be *Arvicola* and *Microtus*, respectively.

Regarding the basal lineages of Arvicolinae, our mitochondrial genome tree showed that North American Ondatrini is the sister group of all other Arvicolinae taxa and that Eurasian Lemmini is the sister group of a clade containing Myodini, Microtini, Arvicolini, Ellobiusini, Lagurini, and Pliomyini, albeit with only moderate support (Supplementary Fig. S4). These results are different from those of Abramson et al. ^24^. based on 13 mitochondrial protein-coding genes, suggesting that Lemmini is the sister group of all other Arvicolinae tribes. In our mitochondrial data analysis, we included RNA genes (two rRNA and twenty-two tRNA genes) in addition to the 13 protein-coding genes, which may be one of the possible reasons for the discordant results. On the other hand, our nuclear tree strongly indicated that Ondatrini branched earlier than Lemmini, lending support to our mitochondrial results, although the nuclear datasets included fewer tribes than the mitochondrial dataset (Fig. 2). In addition, our nuclear data analyses robustly resolved all phylogenetic relationships among genera. In contrast, previous attempts to resolve phylogenetic relationships within the Arvicolinae subfamily using morphological characters, combinations of mitochondrial and nuclear genes, or complete mitochondrial genomes all yielded weakly supported and conflicting topologies^23,24,28,30,31,34^. This demonstrated that the phylogenomic nuclear gene dataset was highly effective for phylogenetic reconstruction within Arvicolinae. Therefore, a comprehensive, robust phylogeny of Arvicolinae requires further phylogenomic analyses of nuclear genes from more taxa in the future.

All the vole species distributed in the HDM region could be placed into two tribes, Myodini and Microtini (Fig. 2 and Supplementary Fig. S4). Tribe Myodini included five genera split into two major lineages. One lineage included *Myodes*, *Alticola* and *Craseomys*. Their species are widespread across the Northern Hemisphere. Another lineage included *Caryomys* and *Eothenomys*. *Caryomys* is mainly distributed in the monsoon areas of Eastern Asia, and *Eothenomys* is mainly distributed in the HDM region. Tribe Microtini contained three HDM genera, *Volemys*, *Proedromys*, and *Neodon*, but these three genera did not form a clade. *Volemys* and *Proedromys* were more closely related and were located near the base of Microtini (Fig. 2 and Supplementary Fig. S4). The genus *Neodon* was deeply nested within the tribe Microtini and was more closely related to the northern China genus *Lasiopodomys*. These phylogenetic relationships implied that the origin of the HDM voles was polyphyletic, possibly resulting from multiple migration events.

### Timing and migration route of HDM vole origination

**Fig. 3:**
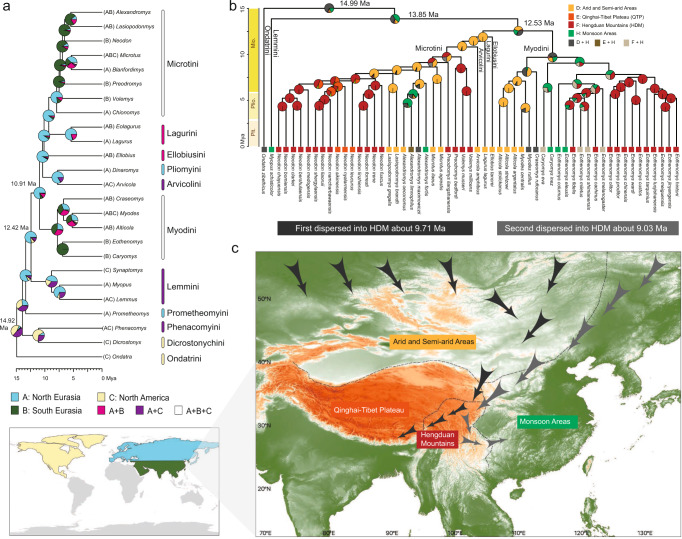
Divergence times and dispersal history of voles. **a** Ancestral range reconstruction of global Arvicolinae based on the time tree from the genus-level mitochondrial genome dataset and the DEC + J model. **b** Reconstruction of the ancestral ranges of the HDM voles and related species based on the time tree of the species-level nuclear dataset and the DEC + J model. The distribution of relevant vole species was divided into four areas: arid and semiarid areas, the Qinghai-Tibet Plateau, the Hengduan Mountains and monsoon areas. Circles on the nodes represent the set of possible ancestral areas, and the color is associated with the area legends. **c** Possible migration routes of voles into the Hengduan Mountains.

Our mitochondrial genome dataset (analyzed at the genus level) and species-level nuclear dataset produced similar divergence time estimates for Arvicolinae evolution (Supplementary Figs. S5 and S6). The major difference between the two estimates was that the mitochondrial times tended to be slightly younger than the nuclear times at shallower nodes. Both mitochondrial and nuclear time trees showed that the most recent common ancestor (MRCA) of extant arvicolids occurred in the middle Miocene at ~14.92 Mya (mitochondrial) or ~14.99 Mya (nuclear) (Fig. 3), immediately after the Miocene Climatic Optimum (17–15 Mya). This time estimate corresponded to the molecular dating results obtained in several previous studies^23,27^ but was much older than those estimated by some other authors (~7 Mya)^24,28^. The MRCA of the tribe Microtini was dated to 9.71 Mya (95% HPD: 8.49–10.99; Supplementary Fig. S6), and the MRCA of the genera *Caryomys* and *Eothenomys* was dated at 9.03 Mya (95% HPD: 7.92–10.1; Supplementary Fig. S6), which corresponded to the origin times of the two major lineages of the HDM voles. Finally, the four vole genera that are largely endemic to the HDM, *Eothenomys*, *Volemys*, *Proedromys* and *Neodon*, originated ~6–7 Mya.

We estimated the historical biogeography of the global Arvicolinae based on the mitochondrial genome dataset (genus-level) using BioGeoBEARS. All models with an additional J parameter that modeled long-distance or “jump” dispersal performed significantly better than the original models (Supplementary Fig. S7), suggesting that long-distance dispersal was a common phenomenon during the diversification of arvicolids. The DEC + J model and the DIVALIKE + J model produced similar ancestral range reconstruction results (Supplementary Fig. S7). Because the oldest known Arvicolinae fossil was found in the Palearctic realm and a fossil record of early arvicolids in North America is lacking, it is generally thought that the common ancestor of Arvicolinae first appeared in Eurasia rather than in North America^35,36^ and migrated from the Palearctic to the Nearctic^24^. However, our biogeographic analyses showed that the common ancestor of extant Arvicolinae most likely first appeared in North America (probability 45.2%) (Fig. 3a). The second probable ancestral area of Arvicolinae was a mixed region of North America and North Eurasia (probability ~ 44%). All these results suggest that the possibility that North America was the region of origin cannot be ruled out, and the migration route of early Arvicolinae might have been from North America to Eurasia.

The mitochondrial genome dataset showed that the common ancestor of all vole species in the HDM came from North Eurasia and dispersed southward thereafter (Fig. 3a). The species-level nuclear dataset provided further information on the origin and migration history of HDM voles (Fig. 3b; See detail results in Supplementary Fig. S8). The common ancestor of the first HDM vole lineage (including *Volemys*, *Proedromys*, and *Neodon*) appeared in arid and semiarid areas of Eurasia (probability ~95%; Supplementary Fig. S8) and entered the HDM region 9.71 Ma (Fig. 3b). The current distribution of this lineage is concentrated in the western part of the HDM region, with some species extending to the QTP. The ancestral area of the other HDM lineage (including *Caryomys* and *Eothenomys*) was estimated to be the monsoon area of Eurasia (probability ~65%; Supplementary Fig. S8). This lineage migrated to the HDM region 9.03 Ma (Fig. 3b). The current distribution of this lineage is concentrated in the eastern part of the HDM region, with some species extending to eastern monsoon areas. Notably, the basal taxa of the two vole lineages (*Proedromys bedfordi* and *Caryomys eva*) were all distributed in the northeastern part of the HDM (see the sample distribution map in Fig. 2 and Supplementary Fig. S9), suggesting that the ancestral voles may have entered the HDM region from the Northeast.

According to these results, we argued that the voles currently distributed in the HDM region likely resulted from two independent colonization events from northern Eurasia. The ancestors of *Volemys*, *Proedromys*, and *Neodon* passed through the arid and semiarid areas of Eurasia and entered the HDM region from the northeast in the late Miocene (~9.71 Mya), after which they migrated southwestward, ultimately reaching the QTP (Fig. 3c). Almost at the same time, the ancestors of *Caryomys* and *Eothenomys* started from the northern part of the monsoon areas of Eurasia and entered the HDM region from the northeast (~9.03 Mya). Their decedents further migrated southeastward, with some species spreading out of the HDM region and finally reaching the southern part of the monsoon areas of Eurasia (Fig. 3c).

### Orogeny promotes the diversification of voles in the HDM

Orogeny creates a variety of environmental conditions, including the generation of climatic niches, new habitats or food resources and dispersal barriers, that promote the speciation of organisms^3,6,37^. The HDM are a geologically young region but possess the highest species richness of voles on a global scale. Is the high species richness of voles related to the recent orogeny in the HDM region?

**Fig. 4:**
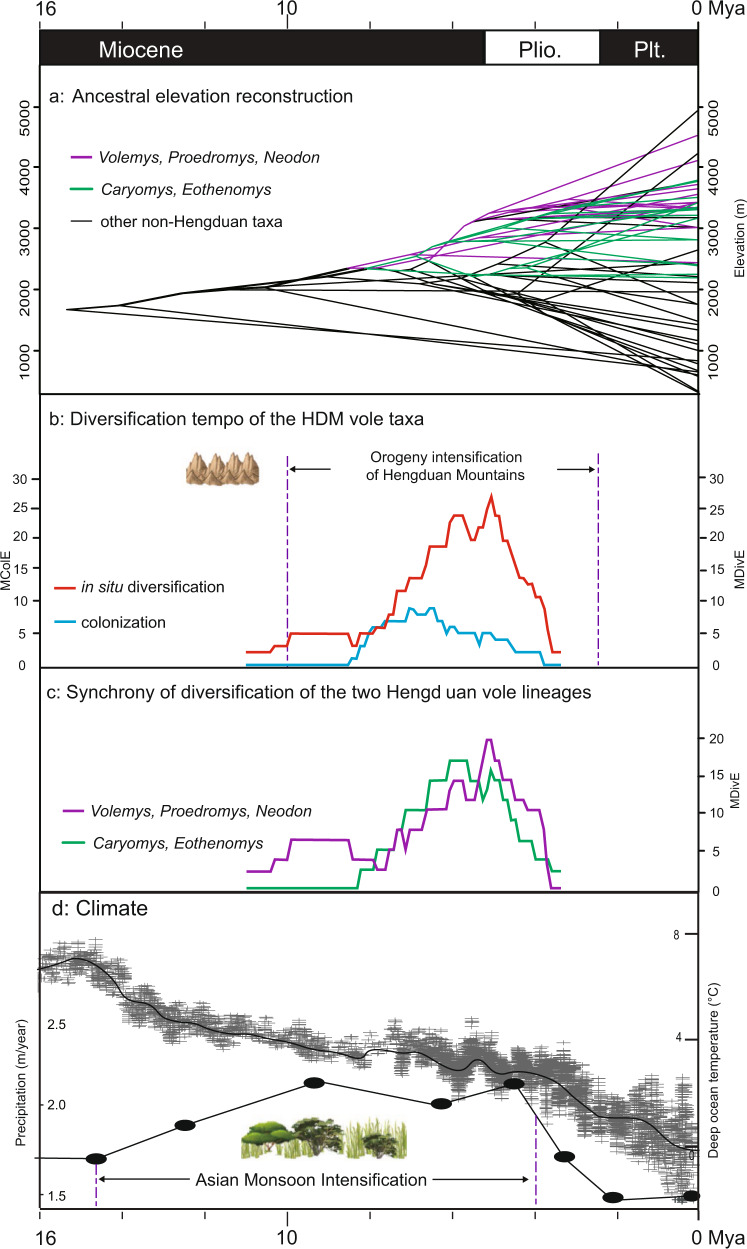
Diversification mode and tempo of voles in the Hengduan Mountains. **a** The reconstruction of ancestral elevation suggests an evolutionary trend of high-elevation adaptation in HDM vole species (green and purple branches) relative to vole species outside the HDM region (black branches). **b** In situ diversification and colonization rates of HDM voles over time (smoothed across 0.1 Ma windows). MDivE = maximal number of observed in situ diversification events per 0.1 Ma. MColE = maximal number of observed colonization events per 0.1 Ma. **c** The in situ diversification rates of western HDM voles (*Volemys*, *Proedromys*, and *Neodon*) and eastern HDM voles (*Caryomys* and *Eothenomys*) are synchronous over time. **d** Global temperature trends and monsoon conditions (indicated by annual precipitation) from the Miocene to the present (modified from Ding et al. ^7^).

To answer this question, we first investigated the evolutionary trend of the elevation adaptation of voles. Our ancestral elevation reconstruction results showed that the ancestors of arvicolids lived in a low-elevation region (1000–2000 m) and that the ancestors of the two HDM vole lineages were distributed in middle elevations (2000–2500 m) (Fig. 4a). During their evolution, the two HDM vole lineages have adapted to higher elevation habitats almost continuously (Fig. 4a). The evolutionary trend of high-elevation adaptation was more obvious in the western HDM lineage (*Volemys*, *Proedromys*, and *Neodon*) (purple lines; Fig. 4a) than in the eastern HDM lineage (*Caryomys* and *Eothenomys*) (green lines; Fig. 4a), in accord with the terrain features of the HDM region, in which the western part is higher than the eastern part. In contrast, the direction of elevation adaptation among vole species outside the HDM region was random, with the species occupying habitats at either higher or lower elevations (black lines; Fig. 4a). These results indicate that the development of vole biodiversity in the HDM occurred via an uplift-driven diversification process.

To further verify this hypothesis, we estimated the speciation mode of all HDM vole species. We identified 44 in situ diversification events in the HDM region and 13 colonization events (Supplementary Fig. S10; Supplementary Table S3). In situ diversification events accounted for over two–thirds of the total speciation events (44/57 = 77.2%), and the rate of in situ diversification was 2–3 times faster than that of colonization (Fig. 4b), suggesting that in situ diversification was the main speciation mode of the HDM voles. The rates of the in situ diversification and colonization of the HDM voles over time showed that the two types of speciation began at similar rates ~10 Mya. After 8 Mya, the rate of in situ diversification accelerated dramatically and reached its peaked rate ~6–5 Mya, while the rate of colonization did not change as greatly (Fig. 4b). Notably, the rates of in situ diversification in the western and eastern vole lineages of the HDM exhibited remarkable synchrony over time (Fig. 4c). The higher diversification rates of voles and the diversification synchronicity of the two vole lineages ~8–4 Mya coincided with the rapid mountain uplift period in the HDM region from the late Miocene to the late Pliocene^38–41^, which strongly suggested a close relationship between the diversification of the HDM voles and the orogenic activity of the HDM.

As part of the continued expansion of QTP orogeny, the HDM underwent recent rapid uplift during the late Miocene, reaching their peak elevation before the late Pliocene^40^. The intensive orogeny of the HDM resulted in extreme ruggedness of the terrain as well as remarkable environmental heterogeneity, creating complex microclimates and fragmented habitat niches^1,42^. The terrain and climate condition changes forced the voles to inhabit narrower regions and smaller elevational ranges, which in turn facilitated the speciation of the high-altitude-adapted voles. At the same time, the Asian monsoon intensified and promoted the growth of plants in the HDM region (Fig. 4d), which provided substantial food resources for rodents^5,7^. We argue that these conditions might together provide new ecological and evolutionary opportunities for voles, leading to the formation of new species.

### Ancient genetic admixture fueled the rapid diversification of voles in the HDM

In addition to environmental factors, speciation can be promoted by biotic factors, such as key traits that allow the exploitation of new niches or gene flow between divergent lineages, leading to faster responses to environmental changes^8,9,43^. We noticed that the species richness of different vole genera in the HDM was highly asymmetrical: *Neodon* (15 species) and *Eothenomys* (17 species) comprised ~85% of the total vole species in the HDM region, while the other three HDM genera (*Volemys*, *Proedromys*, and *Caryomys*) accounted for only 15%. Why do *Neodon* and *Eothenomys* have more species than other HDM genera? Because high-elevation adaptation is a common trait of all HDM voles underlying their continued success in occupying new mountain niches, the high species richness of *Neodon* and *Eothenomys* might be related to gene flow. We wish to unravel the gene flow pattern among vole species of the HDM region to test this hypothesis.

Because the HDM voles are all placed in Myodini and Microtini, we hypothesize that intertribal gene flow occurs between the two tribes. In addition, both Myodini and Microtini contain genera that are endemic to the HDM (HDM genera) and genera distributed outside of the HDM (non-HDM genera). If gene flow plays an important role in the rapid diversification of HDM voles, we expected to observe stronger intertribal gene flow in the HDM genera than in the non-HDM genera. Following this idea, we investigated our species-level nuclear dataset for signals of intertribal gene flow by using Patterson’s *D* test. We performed two separate tests: (A) using Microtini as *P*_3_ group, HDM genera of Myodini as *P*_1_ group and non-HDM genera of Myodini as *P*_2_ group; and (B) using Myodini as *P*_3_ group, HDM genera of Microtini as *P*_1_ group and non-HDM genera of Microtini as *P*_2_ group.

**Fig. 5:**
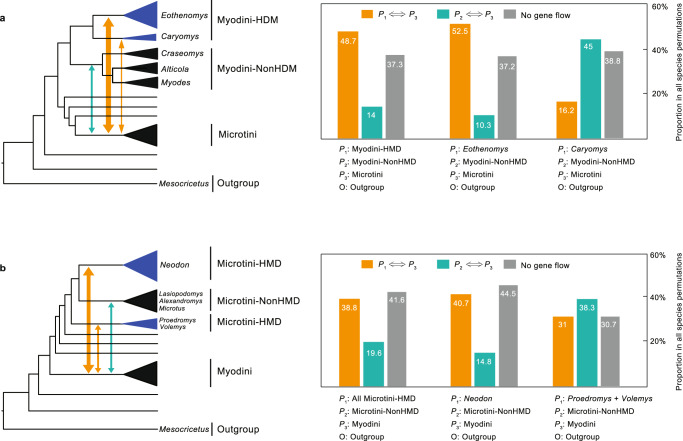
Intertribal gene flow pattern between Microtini and Myodini. A schematic representation of Patterson’s *D* test is provided on the left for each test: HDM genera are indicated in blue, non-HDM genera are indicated in black, and gene flow between lineages is indicated with double-arrowed lines. **a** Gene flow test using Microtini as *P*_*3*_, the non-HDM genera of Myodini as *P*_*2*_, and different components of the HDM genera of Myodini as *P*_*1*_. **b** Gene flow test using Myodini as *P*_*3*_, the non-HDM genera of Microtini as *P*_*2*_, and different components of the HDM genera of Microtini as *P*_*1*_. The histograms to the right of the schematic representation show the percentages of species permutations in which different types of gene flow were detected by Patterson’s *D*-test.

Patterson’s *D* tests were performed on all possible permutations of three species belonging to each of the three test groups using *Mesocricetus auratus* as an outgroup, and the gene flow pattern was interpreted from the number of species permutations in which significant gene flow was detected. In test A, there were a total of 3078 species permutations (Supplementary Table S4), and gene flow was detected in 62.7% of the species permutations (Fig. 5a), showing frequent intertribal gene flow. However, the frequency of gene flow between the HDM genera of Myodini and Microtini and that between the non-HDM genera of Myodini and Microtini were quite different, where the former was ~3.5 times higher than the latter (Fig. 5a). This difference in frequency remained stable when we used only the non-HDM genera or the HDM genera of Microtini as the *P*_3_ group (Supplementary Fig. S11). Notably, for the two HDM genera of Myodini, when only the species-rich *Eothenomys* was used as the *P*_1_ group, the higher gene flow frequency between *P*_1_ and *P*_3_ remained; however, when only species-poor *Caryomys* was used as the *P*_1_ group, the higher gene flow frequency between *P*_1_ and *P*_3_ disappeared (Fig. 5a). These results suggest that the HDM genera of Myodini show more genetic exchange with Microtini voles than the non-HDM genera of Myodini and that species-rich *Eothenomys* presents more frequent intertribal gene flow than species-poor *Caryomys*. Similarly, in test B, when Myodini was used as *P*_3_, and the HDM genera and non-HDM genera of Microtini were used as *P*_1_ and *P*_2_, higher intertribal gene flow frequency in the HDM genera was observed again, and species-rich *Neodon* presented more frequent intertribal gene flow than species-poor *Volemys* and *Proedromys* (Fig. 5b). Because Myodini and Microtini diverged 11–12 million years ago (Fig. 3), the observed intertribal gene flows reflected a kind of ancient genetic admixture among voles.

Based on the observed gene flow patterns, we argue that, to some extent, ancient genetic admixture of different vole lineages increased the rapid diversification of voles in the HDM, which is in line with an admixture variation speciation scenario^9^. This hypothesis explains why *Neodon* and *Eothenomys* have many more species than other HDM genera, although all HDM voles are well suited for mountain habitats. According to admixture variation theory, genetic admixture can instantaneously generate novel genetic combinations from standing genetic variation, facilitating subsequent rapid radiation^8,9^. Compared with standing genetic variation, which gradually arises from new mutations, the admixture of ancient genetic variation generates a genetic variation pool for selection much more rapidly^9,44^. Large amounts of genetic variation increase the potential for phenotypic evolution and extrinsic reproductive isolation, thereby increasing the propensity for ecological speciation given new ecological opportunities^9,45,46^. The role of admixture variation in boosting rapid speciation has previously been acknowledged in many vertebrate groups, such as cichlid fishes^43,47^, narrow-mouthed frogs^48^, and Darwin’s finches^49,50^. The voles of the HDM might likewise have benefitted from such a genetic combinatorial mechanism during their speciation. On the one hand, frequent gene flow provides highly diverse genetic substrates for the speciation of voles in the HDM; on the other hand, the extreme ruggedness of the terrain and the remarkable environmental heterogeneity of the HDM provide an excellent arena in which these admixed genetic materials may give rise to new species. Our results provide a valuable mammalian case supporting the admixture variation speciation theory.

## Conclusions

Using whole-exome capture sequencing, we generated over 6,000 nuclear protein-coding genes per sample and 117 new mitochondrial genomes for 115 Arvicolinae and 6 Cricetinae samples, covering ~100% of the known vole species in the HDM. Based on these data, we produced a robust time-calibrated phylogenetic hypothesis for the voles of the HDM. Our tree is currently the most comprehensive tree available in terms of phylogenetic diversity, taxa, and the number of genes. We found that North American Ondatrini is the sister group of all other arvicolids, implying that Arvicolinae might have originated in North America and later spread to Eurasia. The voles of the HDM comprise two major lineages: the western lineage (*Volemys*, *Proedromys*, and *Neodon*) and the eastern lineage (*Caryomys* and *Eothenomys*). These two vole lineages likely originated from two independent migration events from North Eurasia to the HDM in the late Miocene. The main speciation mode of the HDM voles is in situ diversification. Both the western and eastern vole lineages of the HDM experienced accelerated diversification from 8–4 Mya and have exhibited remarkable synchrony over time. This diversification tempo was in accordance with the rapid mountain uplift of the HDM from the late Miocene to the late Pliocene, showing that the orogenic activity of the HDM played an important role in driving the diversification of the HDM voles. In addition, we identified strong intertribal gene flow between the two lineages of HDM voles, which suggests that ancient genetic admixture might also have fueled the rapid diversification of voles in the HDM. In summary, our findings reveal the evolutionary history and diversification process of voles in the HDM and contribute to a better understanding of how environmental and biotic factors work together to shape the biodiversity of mountain ecosystems.

## Materials and methods

### Taxon sampling and data collection

We conducted multiple field survey and collected 115 vole and lemming specimens (~100% of the known Arvicolinae species in the HDM region^33,51^) at 55 different localities, representing 7 tribes, 16 genera, and 57 species. Six *Cricetulus* samples were collected as outgroup taxa. All rodent samples were captured by using rat traps following the American Society of Mammologist guidelines and the laws and regulations of China for the implementation of the protection of terrestrial wild animals^52,53^. Hypoxia is used as the method of euthanasia of rodent samples in field. Collection permits were approved by Sichuan Forestry Department. Collecting protocols were approved by the Ethics Committee of the Sichuan Academy of Forestry. All specimens were deposited at the Sichuan Academy of Forestry. Detailed information on these samples, such as the collection locality, latitude, longitude, and altitude, is given in Supplementary Table S1.

For each sample, total genomic DNA was extracted from ethanol-preserved tissues (liver, muscle or fur) using a TIANamp Genomic DNA kit (TIANGEN Inc., Beijing, China). Then, 200 ng of genomic DNA was randomly fragmented to sizes of 200–400 bp with NEBNext dsDNA fragmentase (New England Biolabs) and used for DNA library construction with the NEBNext Illumina DNA Library Prep Kit (New England Biolabs). The SureSelect^xt^ Mouse All Exon Kit (Agilent Technologies) was used to capture the coding sequences of each sample. The hybridization enrichment experiments were performed using the SureSelect^xt^ Reagent Kit (G9611A) following the manufacturer’s protocol. After enrichment, the hybridized libraries were captured with Dynabeads MyOne Streptavidin C1 magnetic beads (Invitrogen) and amplified with index primers. The indexed captured libraries were pooled in equal concentrations and sequenced on an Illumina HiSeqX10 sequencer.

### Data processing and dataset construction

We applied a “read mapping” strategy similar to that used by Wang et al. ^54^. to process our sequence capture data. Briefly, for each sample, the sequence reads were aligned to the reference coding DNA sequences (CDSs) of *Mesocricetus auratus* (24,400 CDSs) using BWA^55^ under a stringent setting of -B 6. The alignments were converted to BAM files with SAMtools^56^, and consensus sequences were generated with BCFtools^57^. In this way, we could obtain 24,400 orthologous CDS sequences for each sample. Each CDS orthologous group was aligned using MUSCLE v.3.8^58^ based on codons. From these CDS alignments, we constructed an individual-level nuclear dataset using the following filtering criteria: (i) the percentage of ambiguous bases per CDS must be <0.1%; (ii) the percentage of missing data per CDS must be <60%; (iii) at least 95% of samples must be included per CDS. 6517 CDS alignments passed these criteria and were concatenated. To reduce missing data, we also constructed a species-level nuclear dataset. Based on the data source of the original 24,400 orthologous CDSs for the 122 samples, each orthologous CDS data of all individuals from every nominal species was merged together. Thus, we could obtain 24,400 orthologous CDSs for 61 species. The CDS alignments were generated as described above. The filtering criteria for the species-level nuclear dataset were as follows: (i) the percentage of ambiguous bases per CDS must be <0.1%; (ii) the percentage of missing data per CDS must be <40%; and (iii) 100% of samples must be included for each CDS. Finally, 6078 CDS alignments passed these criteria and were concatenated.

We also constructed a mitochondrial genome dataset. For this purpose, we extracted mitochondrial genomes from our sequencing data for each sample by using MitoZ^59^. To include more Arvicolinae taxa, we searched the GenBank database to collect all published mitochondrial genome sequences of Arvicolinae (stopping in December 2021). When there were more than two mitochondrial genomes for the same species, we retained only representative sequences from different studies in the case of taxonomic revision. Finally, 98 mitochondrial genomes were downloaded and retained for further analysis (their accession numbers are given in Supplementary Table S5). Each mitochondrial gene was aligned using MUSCLE v.3.8^59^ based on either nucleotides or codons depending on its origin, and alignments were refined by GBlocks v.0.91b^60^ with the default settings.

### Phylogenetic analyses

Phylogenetic trees were reconstructed based on maximum likelihood inference using IQ-TREE version 2^61^. IQ-TREE was also used to select the best-fit models and partitioning schemes according to the Bayesian information criterion. For the individual-level nuclear dataset and the species-level nuclear dataset, the best partitioning scheme involved three separate partitions with separate GTR + F + R2 models for the first and second codon positions, GTR + F + R3 models for the third codon positions; for the mitochondrial genome dataset, the best partitioning scheme involved six separate partitions: all tRNAs combined and the first and second codon positions for all protein-coding genes with the GTR + F + R5 model; 12 S rRNA with TIM2 + F + R5; 16 S rRNA with TIM2 + F + R7; and the third codon positions for all protein-coding genes with TIM3 + F + R9. Branch support was estimated by using the ultrafast bootstrap algorithm (UFBoot) embedded in IQ-TREE with 10,000 replicates. The nuclear and mitochondrial genome datasets and detailed IQTREE running results were deposited in the Mendeley Data Repository (https://data.mendeley.com/datasets/mwyj4m963h).

For the individual-level nuclear dataset and species-level nuclear dataset, we also used coalescence-based ASTRAL-II^62^ to estimate the species tree. We used a data binning strategy to improve multispecies coalescent analyses for handling gene trees with weak phylogenetic signals^63,64^. The CDSs of the individual-level nuclear dataset and the species-level nuclear dataset were divided into 200 bins of similar sizes according to their evolutionary rates. Each bin included ~200–300 CDSs. For each data bin, 200 ML bootstrap trees and the final best-scoring ML tree were estimated using RAxML^65^ under the GTR + GAMMA model. These best-scoring ML trees and bootstrapping trees were used as input files for ASTRAL with the option “–i –b” to calculate the final species tree and branch support.

To quantify the genealogical concordance between the resulting trees and the data, we calculated two concordance factors^66^ by using IQ-TREE. The gene concordance factor (gCF) indicates the percentage of decisive gene trees containing a branch, and the site concordance factor (sCF) indicates the percentage of decisive sites supporting a branch in the reference tree. The individual-level nuclear dataset and the species-level nuclear dataset were tested and divided into 200 bins as mentioned above. The concatenated IQ-TREE ML trees were used as reference trees.

### Divergence time analyses

Molecular time estimation was performed based on the mitochondrial genome dataset and the species-level nuclear dataset using MCMCTree in the PAML package^67^. The aim of the mitochondrial analysis was only to provide a time framework for the global Arvicolinae, so we compressed the mitochondrial genome dataset to the genus level, retaining only one or two sequences per genus. Nine calibration points were used, which are presented in Supplementary Table S6. The Arvicolinae-Cricetinae split was constrained at 18–48 Ma. The minimum age of Ondatrini, Lagurini, Ellobiusini, Arvicolini and Lemmini were set to 4.13, 2.5, 2.6, 3.6, and 3.2 Ma. The minimum and maximum boundaries of *Eothenomys*, *Myodes* and *Volemys* were constrained at 2.7–8.1, 3.6–6.08, and 5.3–12.2 Ma. For the minimum and maximum boundaries of all calibration points, the default 2.5% tail probability in MCMCTree was used (soft bound strategy). The root age prior was set to 31 Ma. The mitochondrial dataset was divided into six partitions, and the nuclear dataset was divided into 20 gene bins according to gene evolution rates using a partitioning strategy similar to that applied in the phylogenetic analyses. ML estimates of the branch lengths for each partition were obtained by using the BASEML programs (in PAML) under the GTR + G model. rgene_gamma (overall substitution rate) was set as G (1, 0.27975) or G (1, 24.7497) for the mitochondrial genome dataset or the species-level nuclear dataset, respectively. sigma2_gamma (rate-drift parameter) was set as G (1, 4.5, 1). The independent rate model (clock = 2) was used to specify the prior rate change across the tree. Two independent MCMC runs were conducted. In each run, the first 10 million iterations were discarded as burn-in, after which sampling was conducted every 150 iterations until 100,000 samples were collected. The stationary state and convergence of each run were checked in Tracer^68^.

### Biogeographic analyses

Ancestral range reconstruction was performed by using BioGeoBEARS^69^. First, based on the global distribution pattern of Arvicolinae, we defined three large geographic areas, North America, North Eurasia and South Eurasia, to estimate the biogeographic history of Arvicolinae using the genus-level time tree of the mitochondrial genome dataset. Second, the distribution of the HDM voles and their Eurasian relatives was further subdivided into four geographic regions (Arid and Semiarid Areas, Monsoon Areas, Qinghai-Tibet Plateau and Hengduan Mountains) to estimate the ancestral range of the HDM voles using the time tree of the species-level nuclear dataset. Two models (dispersal-extinction cladogenesis and dispersal-vicariance analysis) and an additional J (long-distance jumping) parameter were tested. In addition, we performed ancestral elevation reconstruction based on the species elevation information and the time tree of the species-level nuclear dataset using the maximum likelihood approach in Phytools^70^.

To infer the speciation mode of vole species in the HDM region, we defined three distribution types: found only in HDMs, found outside of HDMs, or found in both regions. Ancestral range reconstruction analysis was performed on the species-level nuclear dataset using the DEC + J model. Based on the ancestral range estimation result, the biogeographic events of voles in the HDM region were categorized into two types, in situ diversification and colonization. The identification of biogeographic events largely followed the criteria of Xu et al. ^13^. In brief, in an in situ diversification event, the ancestral node and its descendant node are both distributed in the HDM, whereas in a colonization event, the ancestral node is located outside the HDM but its descendant node is distributed in the HDM. Finally, all in situ diversification and colonization events were sliced to calculate their rates over time, defined as the maximal number of colonization events per 0.1 million years and the maximal number of in situ diversification events per 0.1 million years. These rates were calculated by summing potential colonization or in situ diversification events over time using a sliding window of 0.1 Ma based on the divergence time credibility intervals estimated from the species-level nuclear dataset.

### Gene flow analyses

We used Patterson’s *D* test to estimate gene flow between the vole species within the HDM and outside of the HDM. According to the obtained phylogenetic tree, we performed two gene flow tests between Myodini and Microtini. The *D* test requires a four-taxon phylogeny (((*P*_1_, *P*_2_), *P*_3_), O). In the first test, the two HDM genera of tribe Myodini (*Caryomys* and *Eothenomys*) were considered as *P*_1_, the remaining genera of tribe Myodini distributed outside of the HDM (*Alticola*, *Craseomys* and *Myodes*) were considered as *P*_2_, and all species of tribe Microtini were considered as *P*_3_. In the second test, the three HDM genera of tribe Microtini *Neodon*, *Proedromys* and *Volemys* were considered as *P*_1_, the remaining genera of tribe Microtini (*Alexandromys*, *Lasiopodnmys*, and *Microtus*) were considered as *P*_2_, and all species of tribe Myodini were considered as *P*_3_. *Mesocricetus auratus* was used as the outgroup in all tests. For every four-taxon phylogeny in each test, we applied the *D* test exhaustively for all combinations of four species belonging to each of the four groups. For this purpose, we used a custom Python script to extract subsets from the species-level nuclear dataset. These sequence subsets were used by the program *D*_FOIL_^71^ to generate an AB-pattern count file and then calculate *D* statistics by using the default significance cutoff of 0.01.

### Reporting summary

Further information on research design is available in the Nature Research Reporting Summary linked to this article.

## Supplementary information

Supplementary Materials

Description of Additional Supplementary Data

Supplementary Tables

Reporting Summary

## Data Availability

The raw Illumina sequencing data generated in this paper can be downloaded from the NCBI Sequence Read Archive under the BioProject Accession Number PRJNA820500. The extracted CDS sequences for each sample were deposited in the Mendeley Data Repository (Mendeley Data, V1, doi: 10.17632/mwyj4m963h.1).
