# Supplementary material for: Phylogenomics reveals the evolution, biogeography, and diversification history of voles in the Hengduan Mountains
Source: Commun Biol. 2022 Oct 25;5:1124. doi: 10.1038/s42003-022-04108-y (PMC9596468; doi:10.1038/s42003-022-04108-y)
Supplement: Supplementary file 1 — Supplementary Materials [file 42003_2022_4108_MOESM1_ESM.pdf]

Supplementary Materials for

Phylogenomics reveals the evolution, biogeography, and diversification history of voles in the Hengduan Mountains

XiaoYun Wang,[1] Dan Liang,[1] XuMing Wang,[2] MingKun Tang,[2] Yang Liu,[2] ShaoYing Liu,[2]* Peng Zhang[1]*

*Corresponding author. Email: shaoyliu@163.com; zhangp35@mail.sysu.edu.cn.

This PDF file includes:

Fig. S1. The number of CDS sequences of each sample extracted from the whole-exome sequencing data.
Fig. S2. Phylogeny inferred from the species-level nuclear data set (61 species, 6078 genes, 10,788,858 bp).
Fig. S3. The spatial correlation pattern between gCF, sCF and bootstrap values in the two nuclear data sets.
Fig. S4. Mitogenomic phylogeny of Arvicolinae.
Fig. S5. Divergence times estimated from the genus-level mitochrondrial genome data set using MCMCTree.
Fig. S6. Divergence times estimated from the species-level nuclear gene data set using MCMCTree.
Fig. S7. Ancestral range reconstruction results based on the time tree of the genus-level mitochrondrial genome data set.
Fig. S8. Ancestral range reconstruction results based on the time tree of the species-level nuclear data set.
Fig. S9. The collection location map of all specimens used in this study.
Fig. S10 Identification of *in situ* diversification events and colonization events of Hengduan Mountains' voles.
Fig. S11. Inter-tribal gene flow pattern between Microtini and Myodini.
Table S3. Inferred *in situ* diversification events and Colonization events related to the Hengduan Mountains summarized from Figure S8.
Table S5. The information of published complete mitochondrial genomes of Arvicolinae and Cricetinae used in this study.
Table S6. The information of the 9 calibration points used in this study.

Other Supplementary Materials for this manuscript include the following:

Table S1. (Microsoft Excel format) Information of all samples used in this study.
Table S2. (Microsoft Excel format) The detail information of gCF and sCF.
Table S4. (Microsoft Excel format) Detailed results of Patterson's *D* test.

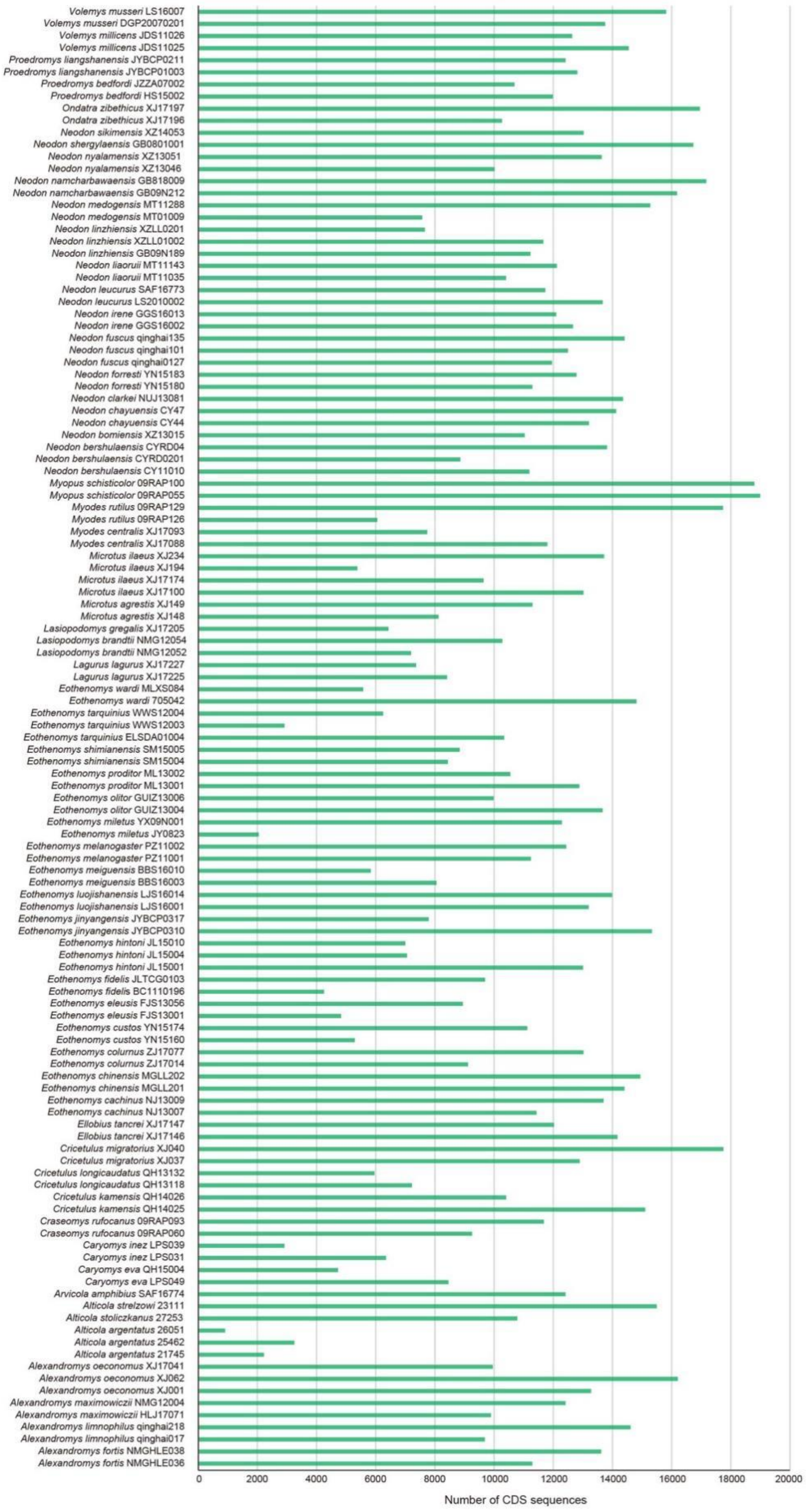

Fig. S1. The number of CDS sequences of each sample extracted from the whole-exome sequencing data.

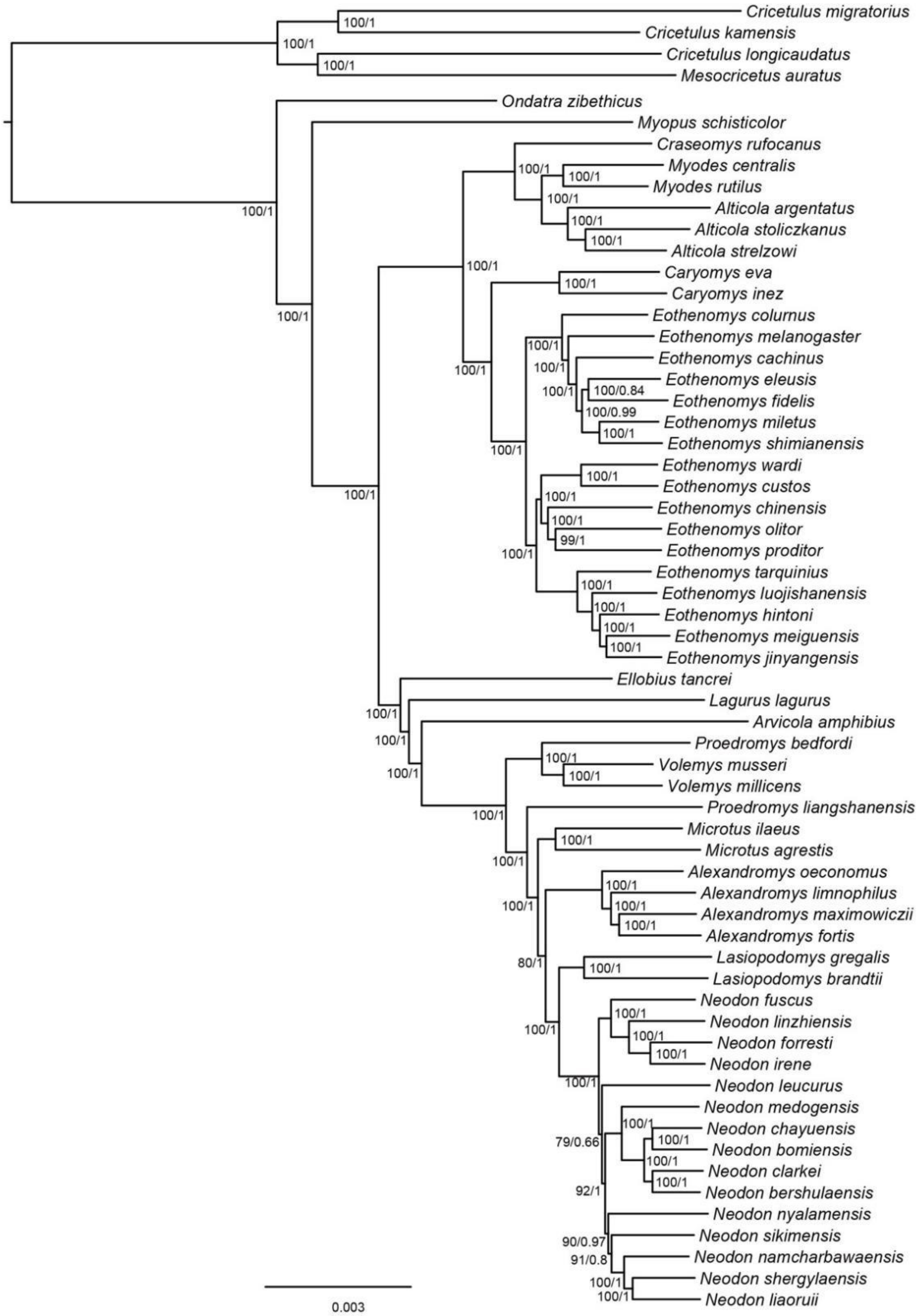

Fig. S2. Phylogeny inferred from the species-level nuclear data set (61 species, 6078 genes, 10,788,858 bp). The tree was inferred by concatenation ML analysis (IQtree) using three partitioned scheme (three codon position partitioning). The coalescent-based species tree was estimated by ASTRAL (the 6078 genes were divided into 200 bins of similar sizes according to their evolutionary rates). The branch support are ultrafast bootstrap values of IQ-TREE (left) and standard bootstrap values of ASTRAL (right).

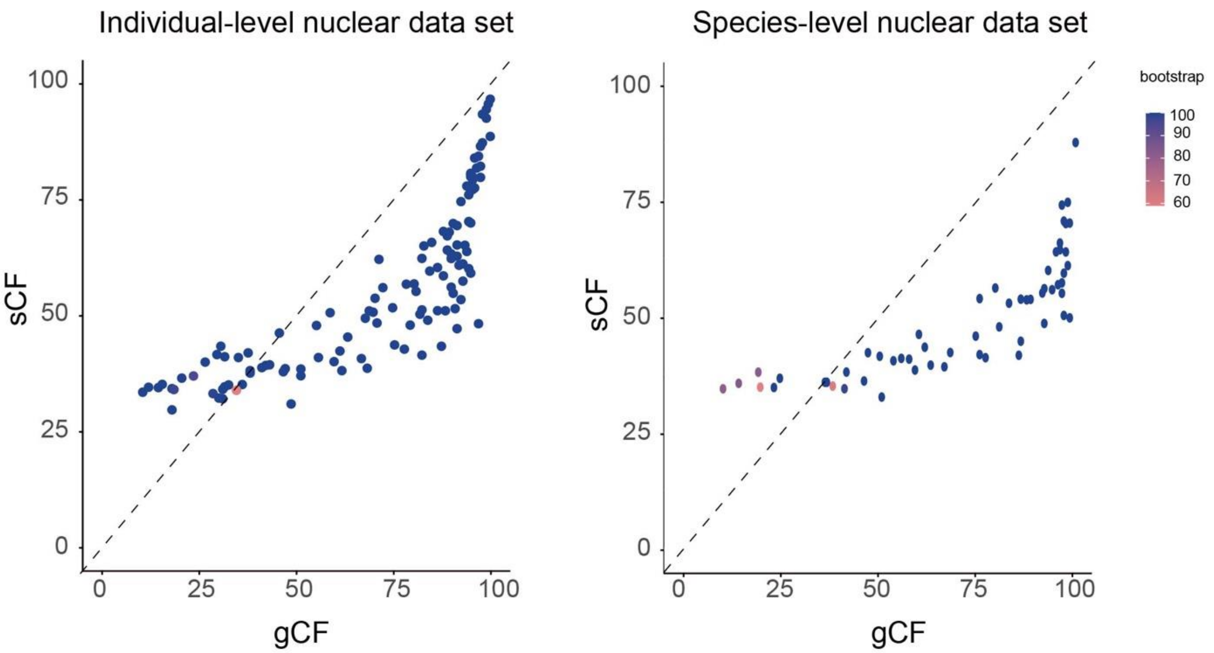

Fig. S3. The spatial correlation pattern between gCF, sCF and bootstrap values in the two nuclear data sets. Each dot represents a branch in the corresponding phylogeny. The detail information of gCF and sCF values are given in table S2.

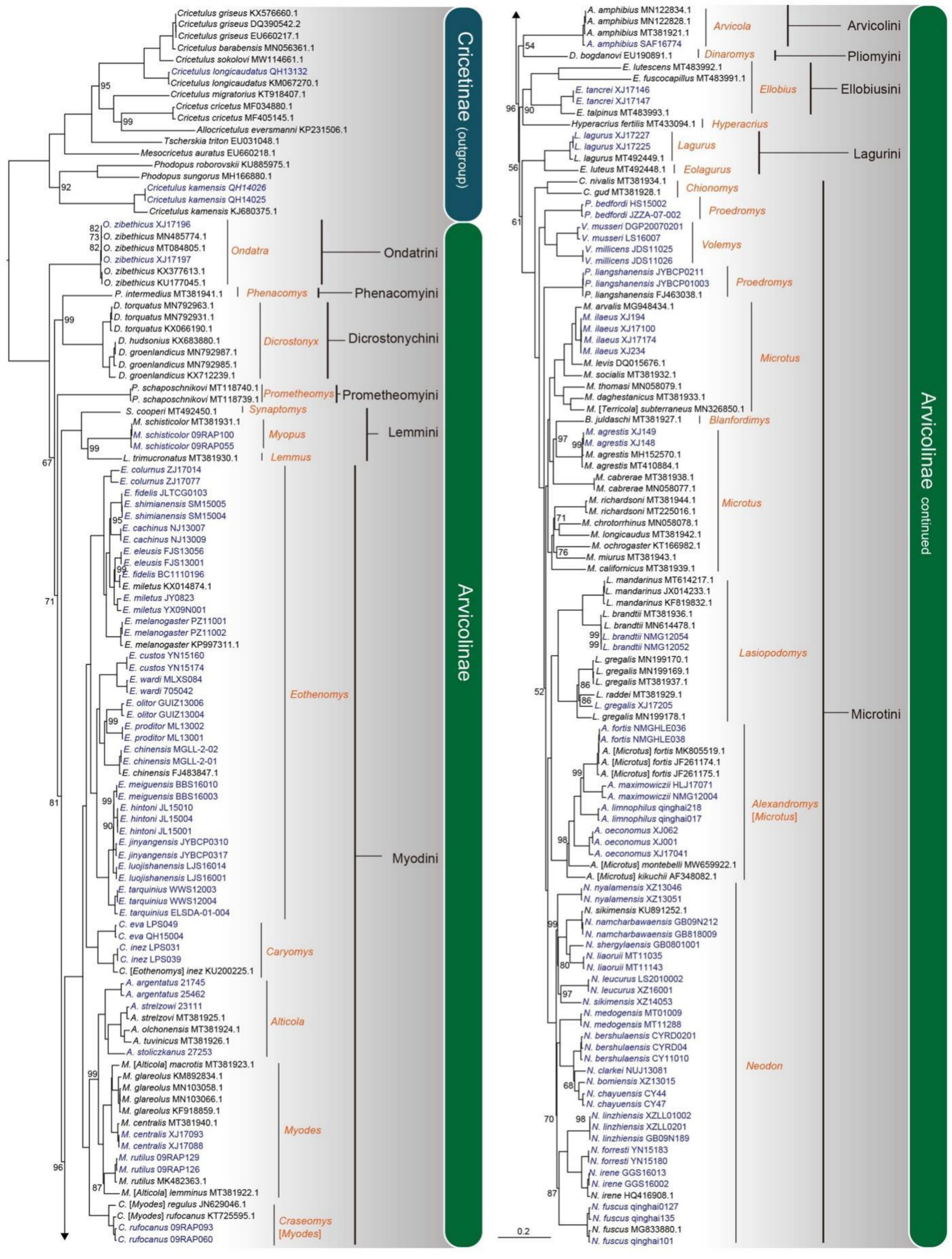

Fig. S4. Mitogenomic phylogeny of Arvicolinae. The tree was inferred from the mitochondrial genome dataset (215 taxa, 15,160 bp) through maximum likelihood analysis in IQ-TREE. Values beside nodes are ultrafast bootstrap (UFBS) values. Nodes without values all show UFBS = 100%. Blue terminals (with sample IDs) are samples that were newly sequenced in this study, while black terminals (with GenBank numbers) come from published mtDNA sequences. The genus names in square brackets are the original genus names of the mtDNA sequences.

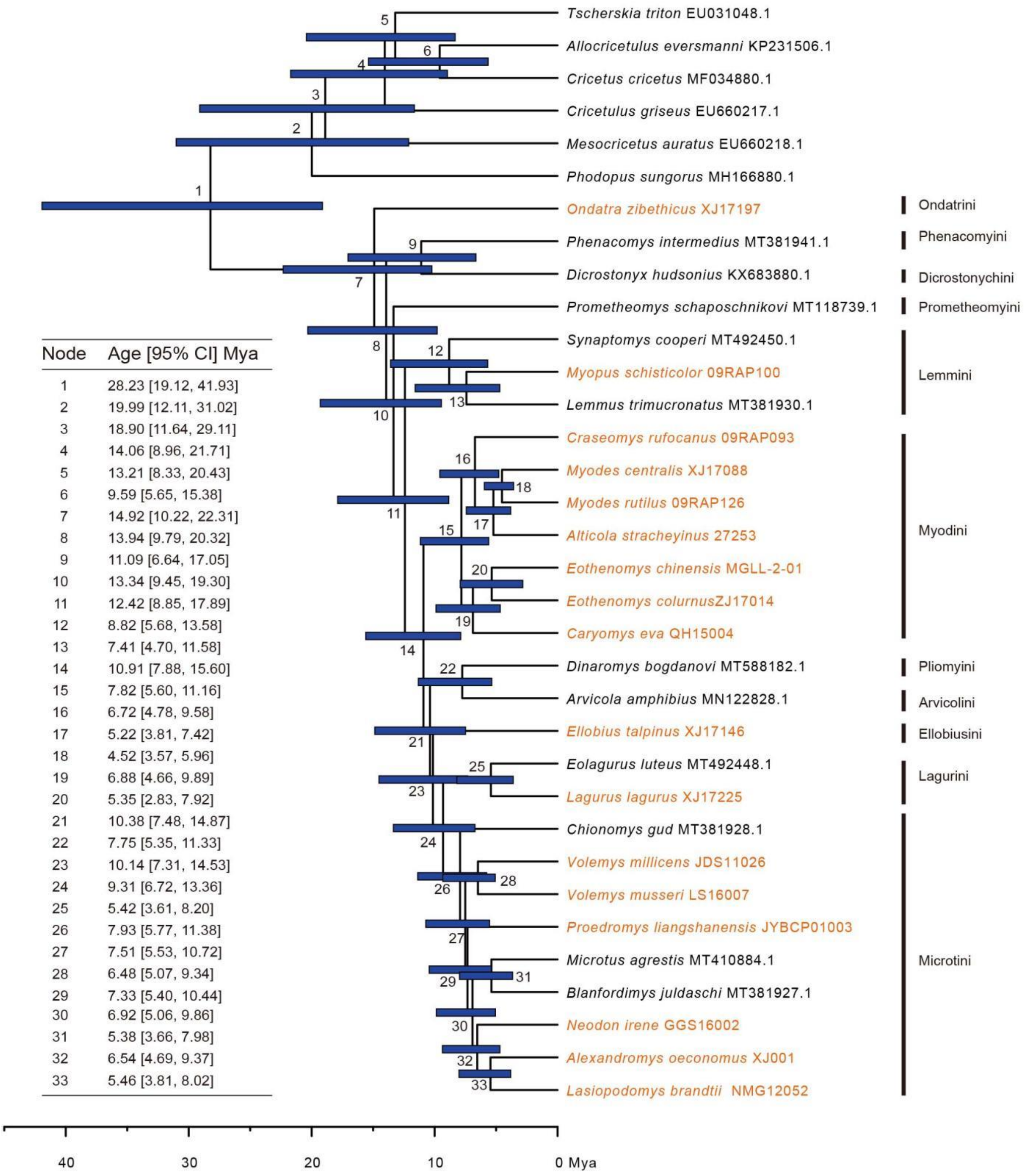

Node	Age [95% CI] Mya
1	28.23 [19.12, 41.93]
2	19.99 [12.11, 31.02]
3	18.90 [11.64, 29.11]
4	14.06 [8.96, 21.71]
5	13.21 [8.33, 20.43]
6	9.59 [5.65, 15.38]
7	14.92 [10.22, 22.31]
8	13.94 [9.79, 20.32]
9	11.09 [6.64, 17.05]
10	13.34 [9.45, 19.30]
11	12.42 [8.85, 17.89]
12	8.82 [5.68, 13.58]
13	7.41 [4.70, 11.58]
14	10.91 [7.88, 15.60]
15	7.82 [5.60, 11.16]
16	6.72 [4.78, 9.58]
17	5.22 [3.81, 7.42]
18	4.52 [3.57, 5.96]
19	6.88 [4.66, 9.89]
20	5.35 [2.83, 7.92]
21	10.38 [7.48, 14.87]
22	7.75 [5.35, 11.33]
23	10.14 [7.31, 14.53]
24	9.31 [6.72, 13.36]
25	5.42 [3.61, 8.20]
26	7.93 [5.77, 11.38]
27	7.51 [5.53, 10.72]
28	6.48 [5.07, 9.34]
29	7.33 [5.40, 10.44]
30	6.92 [5.06, 9.86]
31	5.38 [3.66, 7.98]
32	6.54 [4.69, 9.37]
33	5.46 [3.81, 8.02]

Fig. S5. Divergence times estimated from the genus-level mitochrondrial genome data set using MCMCTree. The terminals in orange color mean that the data of this sample are from this study. Blue bars are 95% confident intervals. Detailed time results for each node are given in the left table.

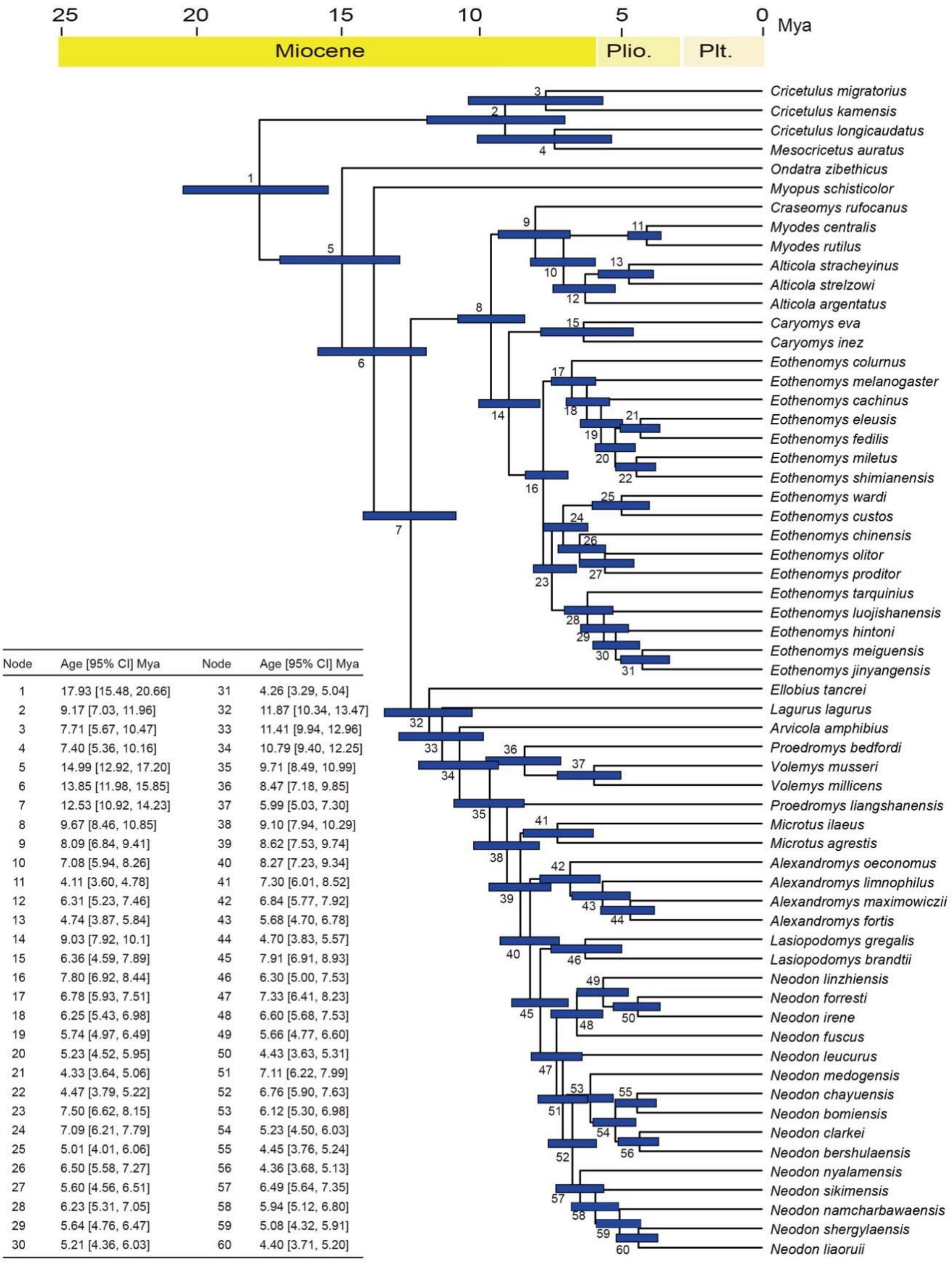

Node	Age [95% CI] Mya	Node	Age [95% CI] Mya
1	17.93 [15.48, 20.66]	31	4.26 [3.29, 5.04]
2	9.17 [7.03, 11.96]	32	11.87 [10.34, 13.47]
3	7.71 [5.67, 10.47]	33	11.41 [9.94, 12.96]
4	7.40 [5.36, 10.16]	34	10.79 [9.40, 12.25]
5	14.99 [12.92, 17.20]	35	9.71 [8.49, 10.99]
6	13.85 [11.98, 15.85]	36	8.47 [7.18, 9.85]
7	12.53 [10.92, 14.23]	37	5.99 [5.03, 7.30]
8	9.67 [8.46, 10.85]	38	9.10 [7.94, 10.29]
9	8.09 [6.84, 9.41]	39	8.62 [7.53, 9.74]
10	7.08 [5.94, 8.26]	40	8.27 [7.23, 9.34]
11	4.11 [3.60, 4.78]	41	7.30 [6.01, 8.52]
12	6.31 [5.23, 7.46]	42	6.84 [5.77, 7.92]
13	4.74 [3.87, 5.84]	43	5.68 [4.70, 6.78]
14	9.03 [7.92, 10.1]	44	4.70 [3.83, 5.57]
15	6.36 [4.59, 7.89]	45	7.91 [6.91, 8.93]
16	7.80 [6.92, 8.44]	46	6.30 [5.00, 7.53]
17	6.78 [5.93, 7.51]	47	7.33 [6.41, 8.23]
18	6.25 [5.43, 6.98]	48	6.60 [5.68, 7.53]
19	5.74 [4.97, 6.49]	49	5.66 [4.77, 6.60]
20	5.23 [4.52, 5.95]	50	4.43 [3.63, 5.31]
21	4.33 [3.64, 5.06]	51	7.11 [6.22, 7.99]
22	4.47 [3.79, 5.22]	52	6.76 [5.90, 7.63]
23	7.50 [6.62, 8.15]	53	6.12 [5.30, 6.98]
24	7.09 [6.21, 7.79]	54	5.23 [4.50, 6.03]
25	5.01 [4.01, 6.06]	55	4.45 [3.76, 5.24]
26	6.50 [5.58, 7.27]	56	4.36 [3.68, 5.13]
27	5.60 [4.56, 6.51]	57	6.49 [5.64, 7.35]
28	6.23 [5.31, 7.05]	58	5.94 [5.12, 6.80]
29	5.64 [4.76, 6.47]	59	5.08 [4.32, 5.91]
30	5.21 [4.36, 6.03]	60	4.40 [3.71, 5.20]

Fig. S6. Divergence times estimated from the species-level nuclear gene data set using MCMCTree. Blue bars are 95% confident intervals. Detailed time results for each node are given in the left table.

A

Model	LnL	Parameters	*d*	*e*	*j*	AIC	*w*
DEC	-47.99	2	4.65	1×10^{-12}	-	99.97	0.08
DEC+J	-45.64	3	4.20	1×10^{-12}	0.08	97.28	0.33
DIVALIKE	-48.61	2	5.00	1×10^{-12}	-	101.21	0.05
DIVALIKE+J	-46.36	3	4.69	1×10^{-12}	0.07	98.72	0.16

Models include DEC (dispersal-extinction cladogenesis), DIVALIKE (dispersal-vicariance analysis), and the same model allowing for founder event speciation (+J). AIC, Akaike Information Criterion; LnL, log-likelihood; *d*, rate of range expansion by adding an area; *e*, rate of range reduction through extirpation in an area; *j*, relative per-event weight of jump dispersal at cladogenesis; *w*, Akaike weights.

B

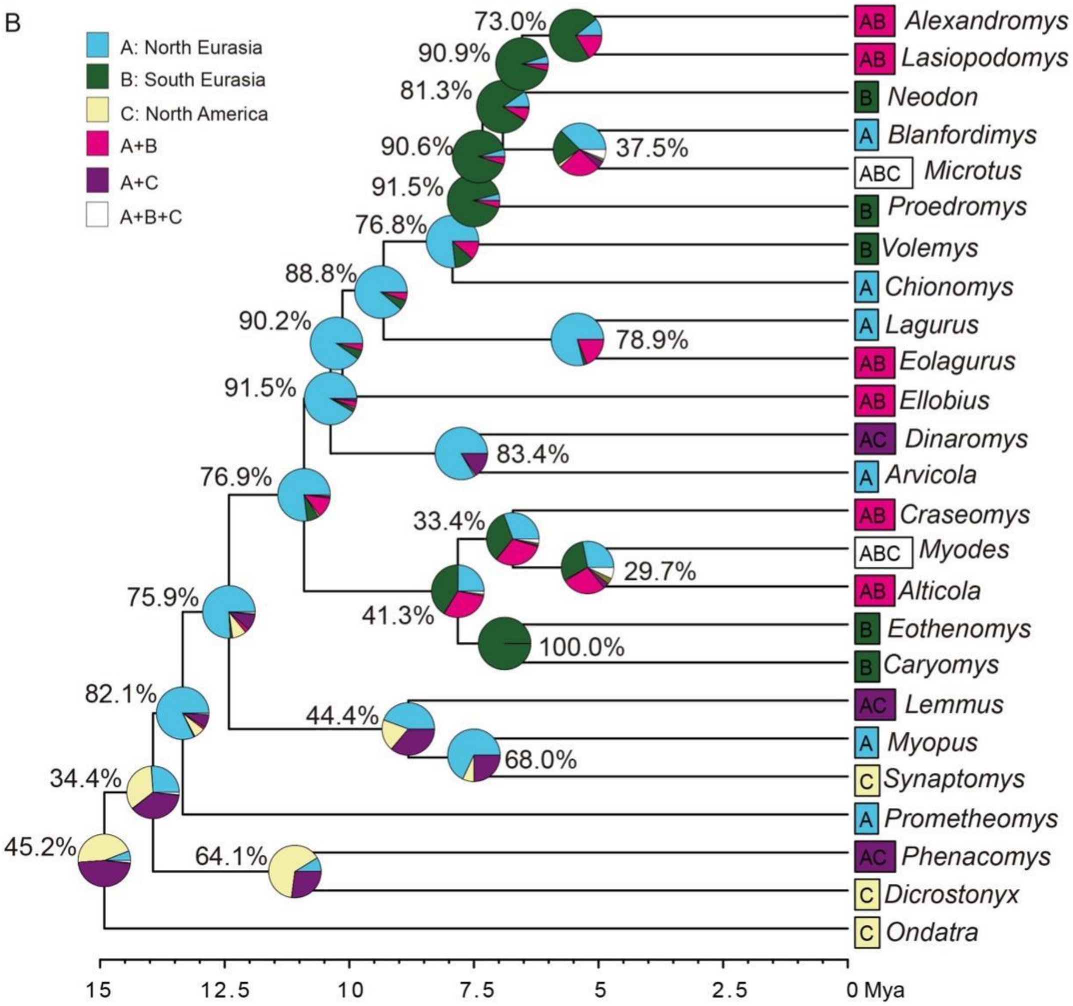

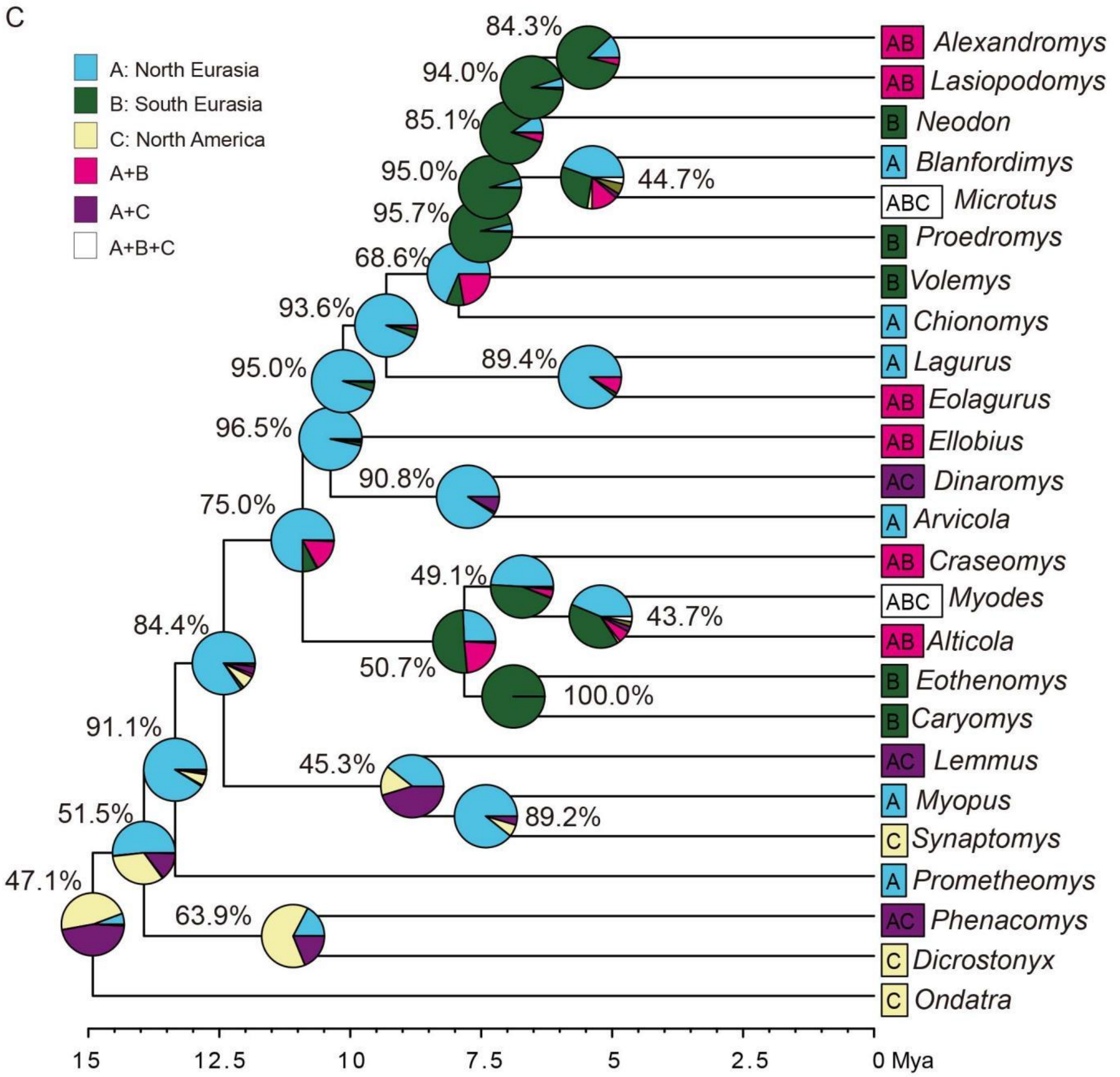

Fig. S7. Ancestral range reconstruction results based on the time tree of the genus-level mitochrondrial genome data set. (A) comparison among models. (B) the result of DEC + J model. (C) the result of DIVALIKE + J model. The probabilities are given next to circles for the most likely ancestral area.

A

Model	LnL	Parameters	d	e	j	AIC	w
DEC	-83.02	2	1.05	1×10^{-12}	-	170.27	0.01
DEC+J	-77.69	3	0.76	1×10^{-12}	0.04	161.83	0.83
DIVALIKE	-93.52	2	1.73	1×10^{-12}	-	191.27	3.37×10^{-12}
DIVALIKE+J	-80.05	3	0.86	1×10^{-12}	0.04	166.56	0.08

Models include DEC (dispersal-extinction cladogenesis), DIVALIKE (dispersal-vicariance analysis), and the same model allowing for founder event speciation (+J). AIC, Akaike Information Criterion; LnL, log-likelihood; *d*, rate of range expansion by adding an area; *e*, rate of range reduction through extirpation in an area; *j*, relative per-event weight of jump dispersal at cladogenesis; *w*, Akaike weights.

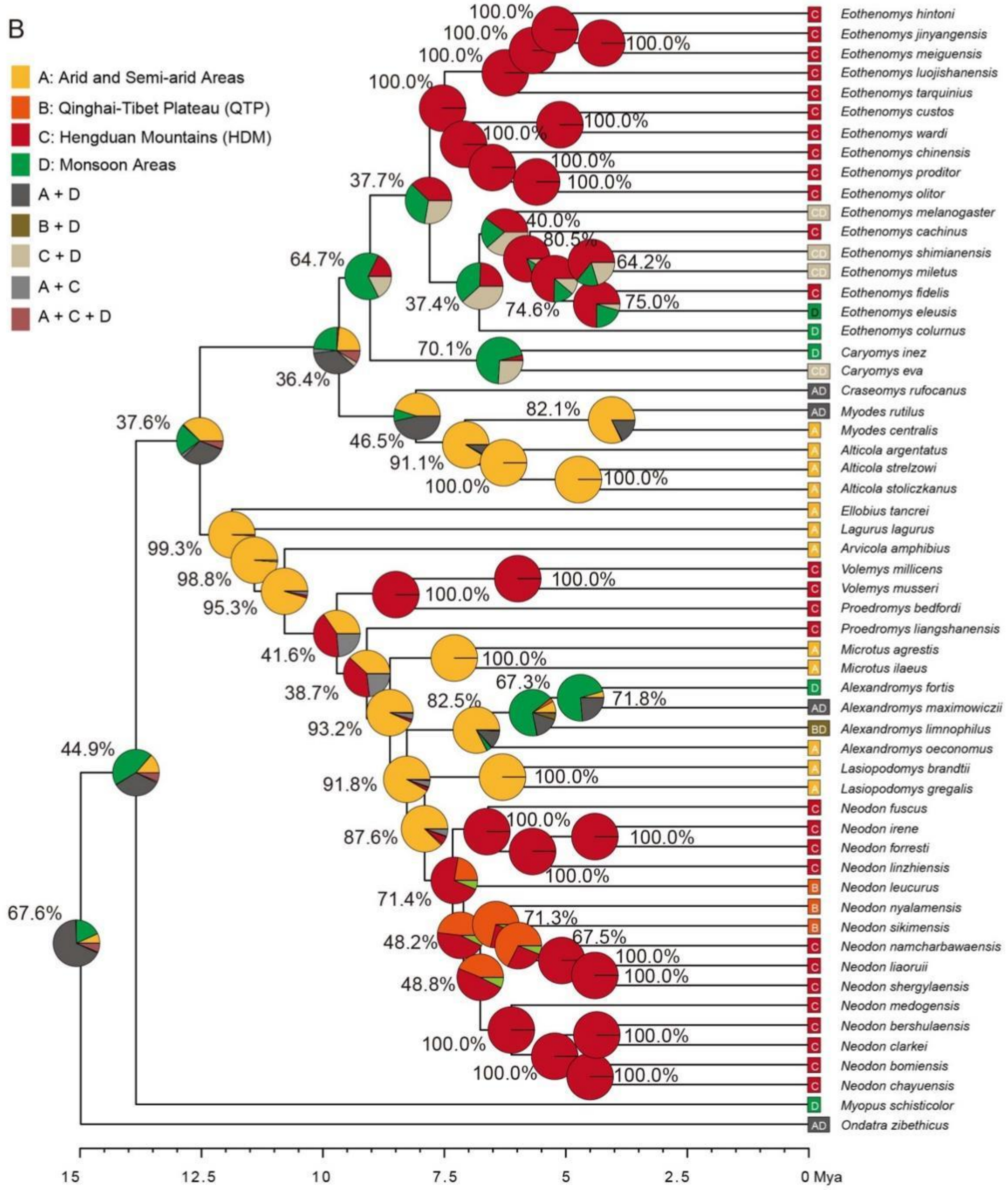

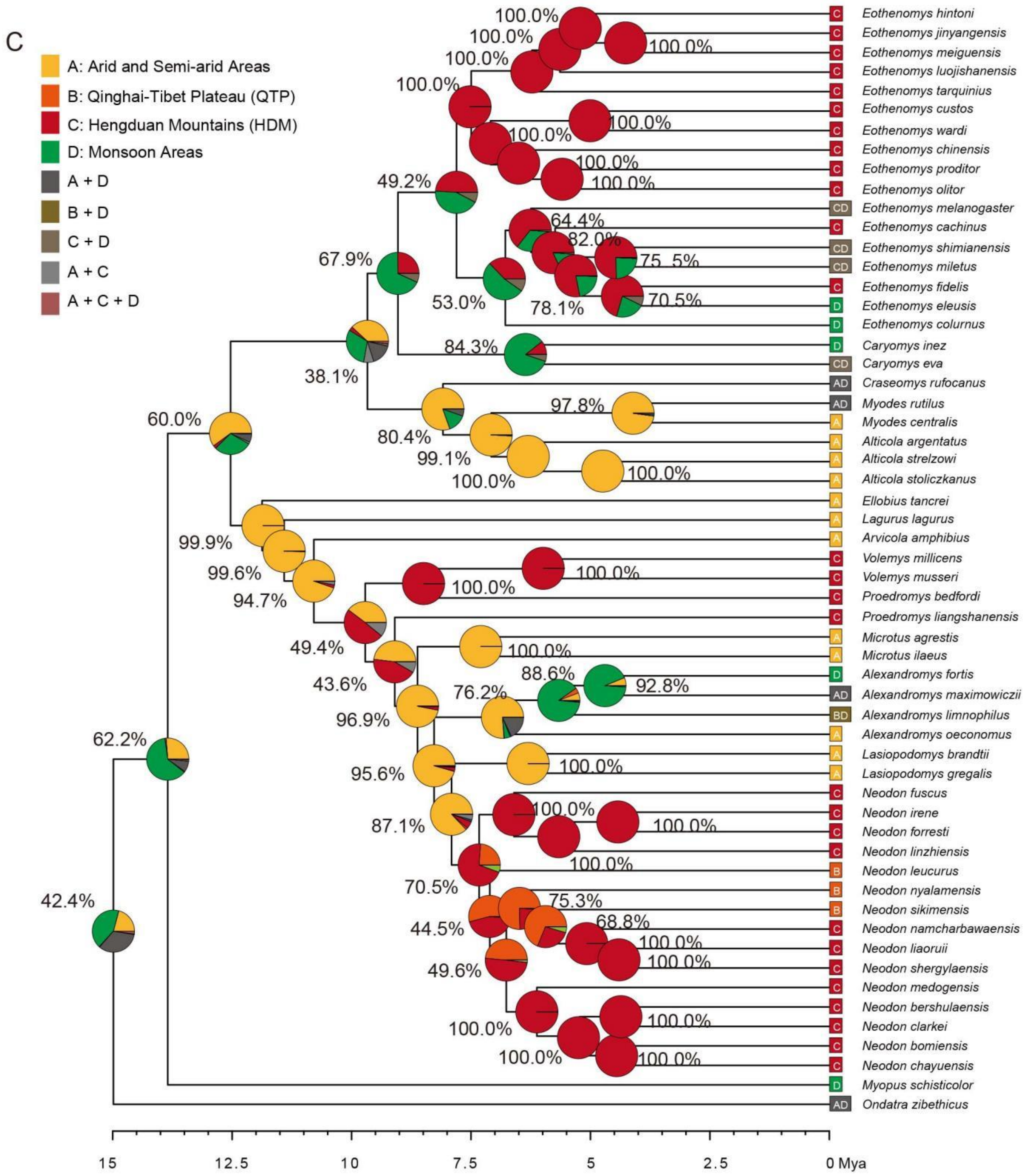

Fig. S8. Ancestral range reconstruction results based on the time tree of the species-level nuclear data set. (A) comparison among models. (B) the result of DEC + J model. (C) the result of DIVALIKE + J model. The probabilities are given next to circles for the most likely ancestral area.

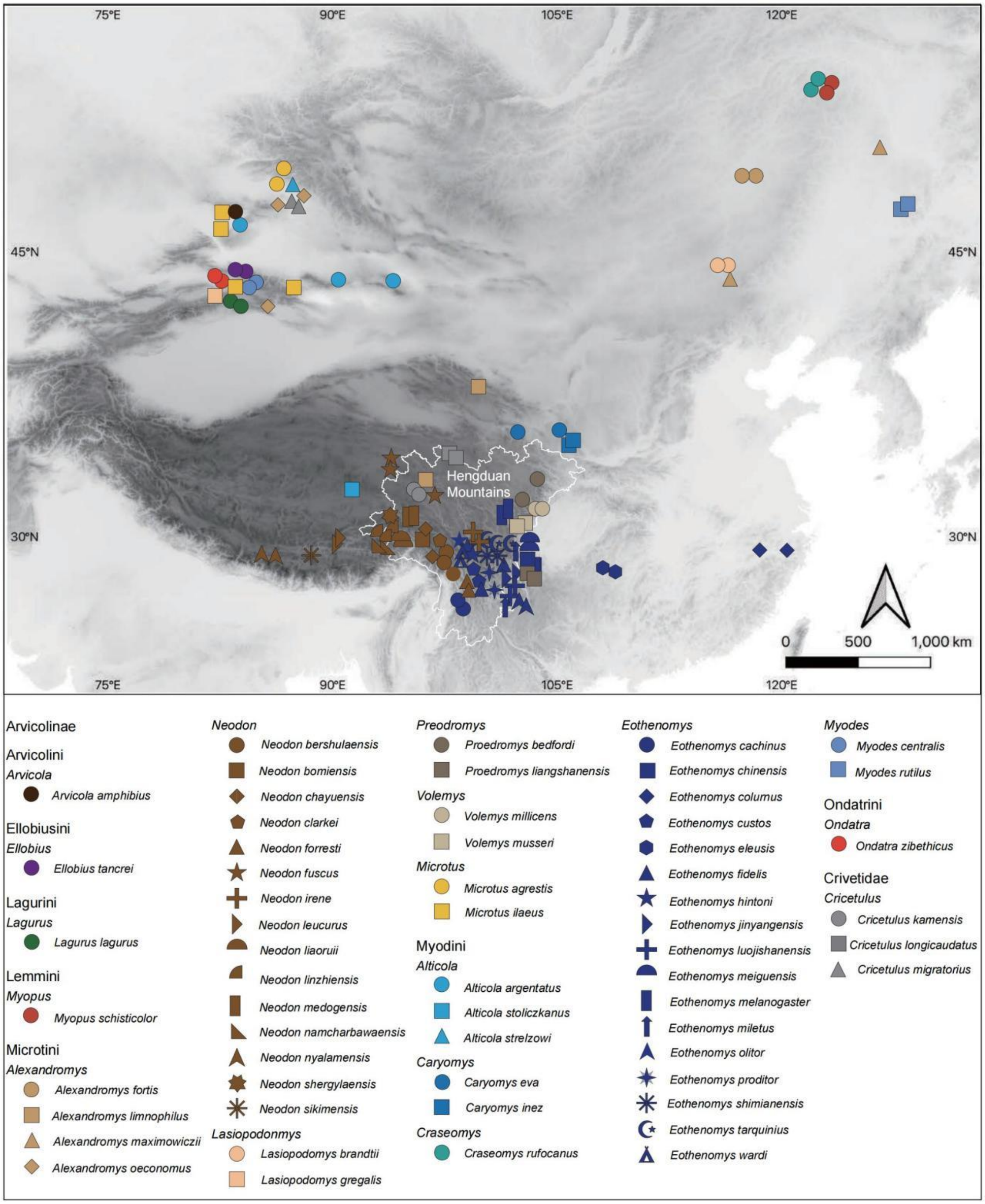

Fig. S9. The collection location map of all specimens used in this study.

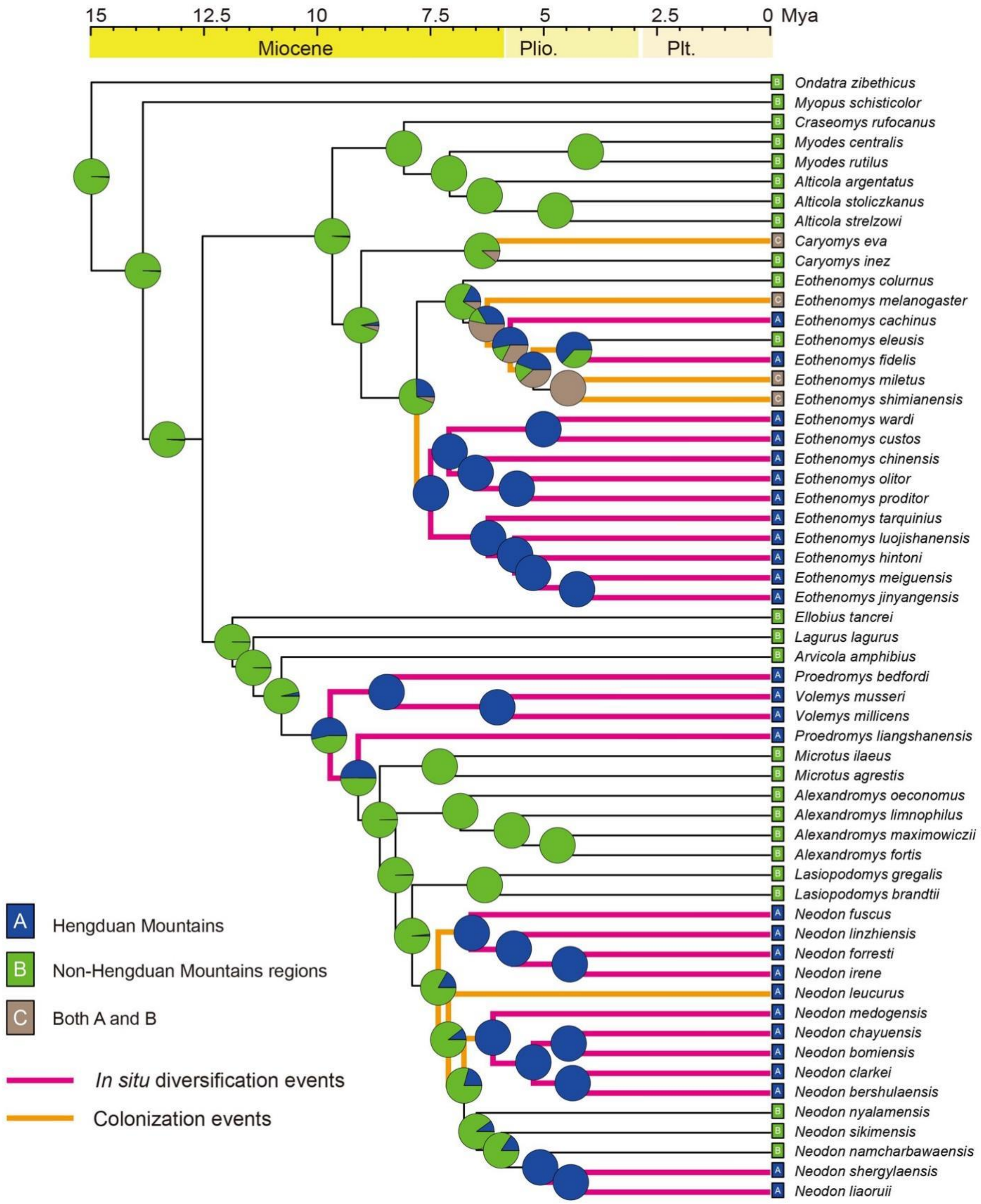

Fig. S10. Identification of *in situ* diversification events and colonization events of Hengduan Mountains' voles. Ancestral range reconstruction was performed with BioGeoBEARS using DEC+j model based on the time tree of the species-level nuclear dataset. Black branches are not related to the Hengduan Mountains diversification.

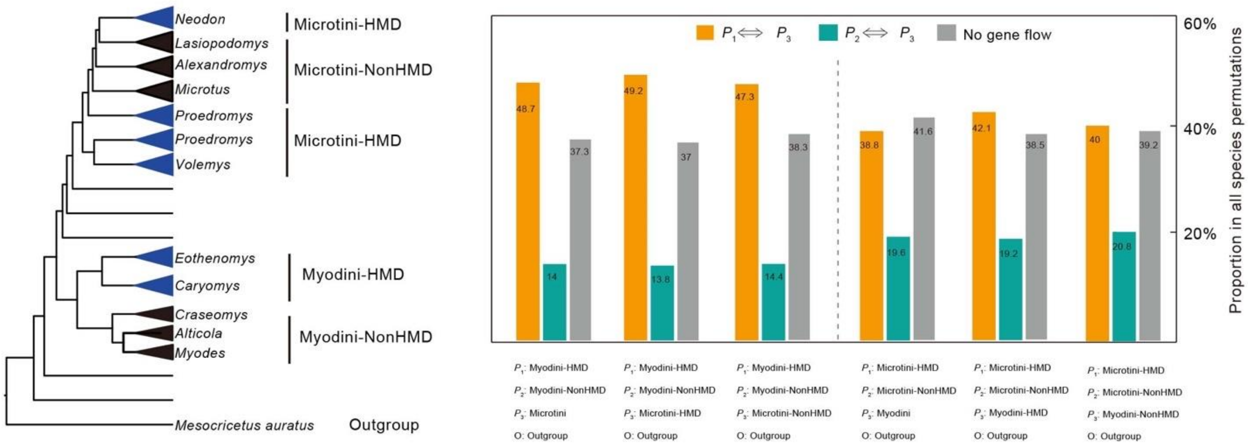

Fig. S11. Inter-tribal gene flow pattern between Microtini and Myodini. The phylogentic tree showing the positions of HDM genera and nonHDM genera of Microtini and Myodini was given in the left. The HDM genera were in blue and the nonHDM genera were in black. Patterson's *D* tests were performed on all possible permutations of three species belonging to each of the three test groups (P_1, P_2, P_3) using *Mesocricetus auratus* as outgroup. The histograms show percentages of species permutations in which different types of gene flow were detected by the Patterson's *D* test.

Table S3. Inferred *in situ* diversification events and Colonization events related to the Hengduan Mountains summarized from Figure S8. The age (mean, upper and lower bounds of the 95% intervals) of respective clade came from the time tree of species-level nuclear data set (Figure S7).

Clades	Mean (Myr)	Upper (Myr)	Lower (Myr)	Events
Eothenomys	7.5	6.62	8.15	*In situ* diversification
Eothenomys	7.09	6.21	7.79	*In situ* diversification
Eothenomys	5.01	4.01	6.06	*In situ* diversification
Eothenomys	6.5	5.58	7.27	*In situ* diversification
Eothenomys	5.6	4.56	6.51	*In situ* diversification
Eothenomys	6.23	5.31	7.05	*In situ* diversification
Eothenomys	5.64	4.76	6.47	*In situ* diversification
Eothenomys	5.21	4.36	6.03	*In situ* diversification
Eothenomys	4.26	3.29	5.04	*In situ* diversification
Eothenomys	7.5	6.62	8.15	*In situ* diversification
Eothenomys	7.09	6.21	7.79	*In situ* diversification
Eothenomys	5.01	4.01	6.06	*In situ* diversification
Eothenomys	6.5	5.58	7.27	*In situ* diversification
Eothenomys	5.6	4.56	6.51	*In situ* diversification
Eothenomys	6.23	5.31	7.05	*In situ* diversification
Eothenomys	5.64	4.76	6.47	*In situ* diversification
Eothenomys	5.21	4.36	6.03	*In situ* diversification
Eothenomys	4.26	3.29	5.04	*In situ* diversification
Proedromys	8.47	7.18	9.85	*In situ* diversification
Proedromys	8.47	7.18	9.85	*In situ* diversification
Volemys	5.99	5.03	7.3	*In situ* diversification
Volemys	5.99	5.03	7.3	*In situ* diversification
Neodon	6.6	5.68	7.53	*In situ* diversification
Neodon	5.66	4.77	6.6	*In situ* diversification
Neodon	4.43	3.63	5.31	*In situ* diversification
Neodon	6.6	5.68	7.53	*In situ* diversification
Neodon	5.66	4.77	6.6	*In situ* diversification
Neodon	4.43	3.63	5.31	*In situ* diversification
Neodon	6.12	5.3	6.98	*In situ* diversification
Neodon	5.23	4.5	6.03	*In situ* diversification
Neodon	4.45	3.76	5.24	*In situ* diversification
Neodon	4.36	3.68	5.13	*In situ* diversification
Neodon	6.12	5.3	6.98	*In situ* diversification
Neodon	5.23	4.5	6.03	*In situ* diversification
Neodon	4.45	3.76	5.24	*In situ* diversification

Neodon	4.36	3.68	5.13	*In situ* diversification
Neodon	5.08	4.23	5.91	*In situ* diversification
Neodon	4.4	3.71	5.2	*In situ* diversification
Neodon	4.4	3.71	5.2	*In situ* diversification
Eothenomys	5.74	4.97	6.49	*In situ* diversification
Eothenomys	4.33	3.64	5.06	*In situ* diversification
Proedromys and *Volemys*	9.71	8.49	10.99	*In situ* diversification
Proedromys	9.71	8.49	10.99	*In situ* diversification
Alexandromys	9.1	7.94	10.29	*In situ* diversification
Caryomys	6.36	4.59	7.89	Colonization
Eothenomys	6.25	5.43	6.98	Colonization
Eothenomys	6.25	5.43	6.98	Colonization
Eothenomys	5.74	4.97	6.49	Colonization
Eothenomys	5.23	4.52	5.95	Colonization
Eothenomys	4.47	3.79	5.22	Colonization
Eothenomys	4.47	3.79	5.22	Colonization
Eothenomys	7.8	6.92	8.44	Colonization
Neodon	7.33	6.41	8.23	Colonization
Neodon	7.33	6.41	8.23	Colonization
Neodon	7.11	6.22	7.99	Colonization
Neodon	7.11	6.22	7.99	Colonization
Neodon	6.76	5.9	7.63	Colonization

Table S5. The information of published complete mitochondrial genomes of Arvicolinae and Cricetinae used in this study.

Subfamily	Genus	Species Name	voucher	Total gene length (bp)	GenBank accession numbers
Arvicolinae	*Alticola*	*Alticola lemminus*	-	16343	MT381922.1
Arvicolinae	*Alticola*	*Alticola macrotis*	-	16355	MT381923.1
Arvicolinae	*Alticola*	*Alticola olchonensis*	-	16403	MT381924.1
Arvicolinae	*Alticola*	*Alticola strelzovi*	-	16423	MT381925.1
Arvicolinae	*Alticola*	*Alticola tuvinicus*	-	15336	MT381926.1
Arvicolinae	*Arvicola*	*Arvicola amphibius*	DM16	16356	MN122828.1
Arvicolinae	*Arvicola*	*Arvicola amphibius*	DM22	16356	MN122834.1
Arvicolinae	*Arvicola*	*Arvicola amphibius*	-	16085	MT381921.1
Arvicolinae	*Blanfordimys*	*Blanfordimys juldaschi*	-	16378	MT381927.1
Arvicolinae	*Chionomys*	*Chionomys gud*	-	16278	MT381928.1
Arvicolinae	*Chionomys*	*Chionomys nivalis*	-	16296	MT381934.1
Arvicolinae	*Dicrostonyx*	*Dicrostonyx torquatus*	UAM:Mamm:84102	16340	KX066190.1
Arvicolinae	*Dicrostonyx*	*Dicrostonyx hudsonius*	-	16338	KX683880.1
Arvicolinae	*Dicrostonyx*	*Dicrostonyx groenlandicus*	-	16341	KX712239.1
Arvicolinae	*Dicrostonyx*	*Dicrostonyx torquatus*	UAM:Mamm:88837	16342	MN792931.1
Arvicolinae	*Dicrostonyx*	*Dicrostonyx torquatus*	UAM:Mamm:89071	16342	MN792963.1
Arvicolinae	*Dicrostonyx*	*Dicrostonyx groenlandicus*	-	16342	MN792985.1
Arvicolinae	*Dicrostonyx*	*Dicrostonyx groenlandicus*	UAM:Mamm:57961	16340	MN792987.1
Arvicolinae	*Dinaromys*	*Dinaromys bogdanovi*	-	16356	MT588182.1
Arvicolinae	*Ellobius*	*Ellobius fuscocapillus*	4907	16388	MT483991.1
Arvicolinae	*Ellobius*	*Ellobius lutescens*	4905	16540	MT483992.1
Arvicolinae	*Ellobius*	*Ellobius talpinus*	1924	16367	MT483993.1
Arvicolinae	*Eolagurus*	*Eolagurus luteus*	-	16354	MT492448.1
Arvicolinae	*Eothenomys*	*Eothenomys chinensis*	-	16362	FJ483847.1
Arvicolinae	*Eothenomys*	*Eothenomys melanogaster*	-	16331	KP997311.1
Arvicolinae	*Eothenomys*	*Eothenomys inez*	301	16354	KU200225.1
Arvicolinae	*Eothenomys*	*Eothenomys miletus*	-	16344	KX014874.1
Arvicolinae	*Hyperacrius*	*Hyperacrius fertilis*	-	16341	MT433094.1
Arvicolinae	*Lagurus*	*Lagurus lagurus*	-	16362	MT492449.1
Arvicolinae	*Lasiopodomys*	*Lasiopodomys mandarinus*	-	16367	JX014233.1
Arvicolinae	*Lasiopodomys*	*Lasiopodomys mandarinus*	-	16375	KF819832.1
Arvicolinae	*Lasiopodomys*	*Lasiopodomys gregalis*	MG2	16292	MN199169.1
Arvicolinae	*Lasiopodomys*	*Lasiopodomys gregalis*	MG1	16294	MN199170.1
Arvicolinae	*Lasiopodomys*	*Lasiopodomys gregalis*	MI1105	16114	MN199178.1
Arvicolinae	*Lasiopodomys*	*Lasiopodomys brandtii*	-	16557	MN614478.1

Arvicolinae	*Lasiopodomys*	*Lasiopodomys raddei*	-	16317	MT381929.1
Arvicolinae	*Lasiopodomys*	*Lasiopodomys brandtii*	-	16374	MT381936.1
Arvicolinae	*Lasiopodomys*	*Lasiopodomys gregalis*	-	16297	MT381937.1
Arvicolinae	*Lasiopodomys*	*Lasiopodomys mandarinus*	LM01	16562	MT614217.1
Arvicolinae	*Lemmus*	*Lemmus trimucronatus*	-	16344	MT381930.1
Arvicolinae	*Microtus*	*Microtus kikuchii*	-	16312	AF348082.1
Arvicolinae	*Microtus*	*Microtus levis*	-	16283	DQ015676.1
Arvicolinae	*Microtus*	*Microtus fortis fortis*	-	16310	JF261174.1
Arvicolinae	*Microtus*	*Microtus fortis calamorum*	-	16310	JF261175.1
Arvicolinae	*Microtus*	*Microtus ochrogaster*	-	16292	KT166982.1
Arvicolinae	*Microtus*	*Microtus arvalis*	15-006	16285	MG948434.1
Arvicolinae	*Microtus*	*Microtus agrestis*	-	16297	MH152570.1
Arvicolinae	*Microtus*	*Microtus fortis pelliceus*	MD2018144	16310	MK805519.1
Arvicolinae	*Microtus*	*Microtus cabrerae*	-	16331	MN058077.1
Arvicolinae	*Microtus*	*Microtus chrotorrhinus*	-	16297	MN058078.1
Arvicolinae	*Microtus*	*Microtus thomasi*	-	16295	MN058079.1
Arvicolinae	*Microtus*	*Microtus richardsoni*	-	16285	MT225016.1
Arvicolinae	*Microtus*	*Microtus socialis*	-	16339	MT381932.1
Arvicolinae	*Microtus*	*Microtus daghestanicus*	-	16297	MT381933.1
Arvicolinae	*Microtus*	*Microtus cabrerae*	-	16327	MT381938.1
Arvicolinae	*Microtus*	*Microtus californicus*	-	16299	MT381939.1
Arvicolinae	*Microtus*	*Microtus longicaudus*	-	16303	MT381942.1
Arvicolinae	*Microtus*	*Microtus miurus*	-	16294	MT381943.1
Arvicolinae	*Microtus*	*Microtus richardsoni*	-	16286	MT381944.1
Arvicolinae	*Microtus*	*Microtus agrestis*	DM50d	16296	MT410884.1
Arvicolinae	*Microtus*	*Microtus montebelli*	HUA1900522	16307	MW659922.1
Arvicolinae	*Myodes*	*Myodes regulus*	-	16379	JN629046.1
Arvicolinae	*Myodes*	*Myodes glareolus*	-	16353	KF918859.1
Arvicolinae	*Myodes*	*Myodes glareolus*	558	16354	KM892834.1
Arvicolinae	*Myodes*	*Myodes rufocanus*	-	16487	KT725595.1
Arvicolinae	*Myodes*	*Myodes rutilus*	-	16295	MK482363.1
Arvicolinae	*Myodes*	*Myodes glareolus*	733	16354	MN103058.1
Arvicolinae	*Myodes*	*Myodes glareolus*	1174	16353	MN103066.1
Arvicolinae	*Myodes*	*Myodes centralis*	-	16350	MT381940.1
Arvicolinae	*Myopus*	*Myopus schisticolor*	-	16339	MT381931.1
Arvicolinae	*Neodon*	*Neodon irene*	-	16367	HQ416908.1
Arvicolinae	*Neodon*	*Neodon sikimensis*	-	16330	KU891252.1
Arvicolinae	*Neodon*	*Neodon fuscus*	-	16328	MG833880.1
Arvicolinae	*Ondatra*	*Ondatra zibethicus*	-	16348	KU177045.1
Arvicolinae	*Ondatra*	*Ondatra zibethicus*	-	16348	KX377613.1
Arvicolinae	*Ondatra*	*Ondatra zibethicus*	-	16351	MN485774.1

Arvicolinae	*Ondatra*	*Ondatra zibethicus*	-	16350	MT084805.1
Arvicolinae	*Phenacomys*	*Phenacomys intermedius*	-	16336	MT381941.1
Arvicolinae	*Proedromys*	*Proedromys liangshanensis*	-	16296	FJ463038.1
Arvicolinae	*Prometheomys*	*Prometheomys schaposchnikovi*	PrScTR1	16284	MT118739.1
Arvicolinae	*Prometheomys*	*Prometheomys schaposchnikovi*	PrScTR2	16284	MT118740.1
Arvicolinae	*Synaptomys*	*Synaptomys cooperi*	-	16346	MT492450.1
Arvicolinae	*Terricola*	*Terricola subterraneus*	-	16296	MN326850.1
Cricetinae	*Allocricetulus*	*Allocricetulus eversmanni*	-	16282	KP231506.1
Cricetinae	*Cricetulus*	*Cricetulus griseus*	-	16284	DQ390542.2
Cricetinae	*Cricetulus*	*Cricetulus griseus*	-	16283	EU660217.1
Cricetinae	*Cricetulus*	*Cricetulus kamensis*	-	16270	KJ680375.1
Cricetinae	*Cricetulus*	*Cricetulus longicaudatus*	-	16302	KM067270.1
Cricetinae	*Cricetulus*	*Cricetulus migratorius*	-	16246	KT918407.1
Cricetinae	*Cricetulus*	*Cricetulus griseus*	-	16283	KX576660.1
Cricetinae	*Cricetulus*	*Cricetulus barabensis*	-	16282	MN056361.1
Cricetinae	*Cricetulus*	*Cricetulus sokolovi*	-	16292	MW114661.1
Cricetinae	*Cricetus*	*Cricetus cricetus*	-	16263	MF034880.1
Cricetinae	*Cricetus*	*Cricetus cricetus*	-	16263	MF405145.1
Cricetinae	*Mesocricetus*	*Mesocricetus auratus*	-	16264	EU660218.1
Cricetinae	*Phodopus*	*Phodopus roborovskii*	-	16273	KU885975.1
Cricetinae	*Phodopus*	*Phodopus sungorus*	XWZ24	16346	MH166880.1
Cricetinae	*Tscherskia*	*Tscherskia triton*	-	16488	EU031048.1

Table S6. The information of the 9 calibration points used in this study.

Node to be calibrated	Fossils	Minimum	Maximum	References
root, Arvicolinae-Cricetinae split	-	18	48	http://timetree.org
Ondatrini (stem)	*Pliopotamys minor*	4.13	-	Abramson et al., 2021
Lagurini (stem)	*Prolagurus pannonicus*<br>*Eolagurus argyropuloi - simplicidens*<br>*Lagurodon*	2.5	-	Abramson et al., 2021
Ellobiusini (stem)	*Ellobius primigenius*	2.6	-	Abramson et al., 2021
Arvicolini (stem)	*Arvicola amphibius*	3.6	-	https://paleobiodb.org/
Lemmini (stem)	*Lemmus europaeus*	3.2	-	Abramson et al., 2021
Eothenomys (crown)	-	2.7	8.1	Hu et al., 2021
Myodes (crown)	-	3.6	6.08	Hu et al., 2021
Volemys (crown)	-	5.3	12.2	Hu et al., 2021

Abramson N I, Semyon B, Bondareva O V, et al. Mitochondrial genome phylogeny of voles and lemmings (Rodentia: Arvicolinae): evolutionary and taxonomic implications. PLoS ONE, 2021, 16(11): e0248198.
Hu Y, Fan H, Chen Y, et al. Spatial patterns and conservation of genetic and phylogenetic diversity of wildlife in China. Science Advances, 2021, 7(4): eabd5725.
