# Supplementary material for: Phylogenomics reveals the evolution, biogeography, and diversification history of voles in the Hengduan Mountains
Source: Commun Biol. 2022 Oct 25;5:1124. doi: 10.1038/s42003-022-04108-y (PMC9596468; doi:10.1038/s42003-022-04108-y)
Supplement: Supplementary file 2 — Description of Additional Supplementary Data [file 42003_2022_4108_MOESM2_ESM.pdf]

Description of Additional Supplementary Files

File name: Supplementary_Table.xlsx

Description:

This EXCEL file includes:

Table S1. (Microsoft Excel format) Information of all samples used in this study.

Table S2. (Microsoft Excel format) The detail information of gCF and sCF.

Table S4. (Microsoft Excel format) Detailed results of Patterson's D test.
