# Supplementary material for: Phylogenomics reveals the evolution, biogeography, and diversification history of voles in the Hengduan Mountains
Source: Commun Biol. 2022 Oct 25;5:1124. doi: 10.1038/s42003-022-04108-y (PMC9596468; doi:10.1038/s42003-022-04108-y)
Supplement: Supplementary file 4 — Reporting Summary [file 42003_2022_4108_MOESM4_ESM.pdf]

nature portfolio

Corresponding author(s): Peng Zhang

Last updated by author(s): Peng Zhang

Reporting Summary

Nature Portfolio wishes to improve the reproducibility of the work that we publish. This form provides structure for consistency and transparency in reporting. For further information on Nature Portfolio policies, see our Editorial Policies and the Editorial Policy Checklist.

Statistics

For all statistical analyses, confirm that the following items are present in the figure legend, table legend, main text, or Methods section.

n/a	Confirmed	
☒	☐	The exact sample size (*n*) for each experimental group/condition, given as a discrete number and unit of measurement
☒	☐	A statement on whether measurements were taken from distinct samples or whether the same sample was measured repeatedly
☒	☐	The statistical test(s) used AND whether they are one- or two-sided<br>*Only common tests should be described solely by name; describe more complex techniques in the Methods section.*
☒	☐	A description of all covariates tested
☒	☐	A description of any assumptions or corrections, such as tests of normality and adjustment for multiple comparisons
☐	☒	A full description of the statistical parameters including central tendency (e.g. means) or other basic estimates (e.g. regression coefficient) AND variation (e.g. standard deviation) or associated estimates of uncertainty (e.g. confidence intervals)
☒	☐	For null hypothesis testing, the test statistic (e.g. *F*, *t*, *r*) with confidence intervals, effect sizes, degrees of freedom and *P* value noted<br>*Give P values as exact values whenever suitable.*
☐	☒	For Bayesian analysis, information on the choice of priors and Markov chain Monte Carlo settings
☒	☐	For hierarchical and complex designs, identification of the appropriate level for tests and full reporting of outcomes
☒	☐	Estimates of effect sizes (e.g. Cohen's *d*, Pearson's *r*), indicating how they were calculated

Our web collection on statistics for biologists contains articles on many of the points above.

Software and code

Policy information about availability of computer code

Data collection	For sequence capture data, we used BWA, SAMtools, and BCFtools to obtain all orthologous coding DNA sequences.
Data analysis	For all orthologous coding DNA sequences, we used IQ-TREE version 2, ASTRAL-II and RAxML to preform phylogenetic analyses. Molecular time estimation used PAML package. Biogeographic analyses used BioGeoBEARS. Gene flow analyses used DFOIL.

For manuscripts utilizing custom algorithms or software that are central to the research but not yet described in published literature, software must be made available to editors and reviewers. We strongly encourage code deposition in a community repository (e.g. GitHub). See the Nature Portfolio guidelines for submitting code & software for further information.

Data

Policy information about availability of data

All manuscripts must include a data availability statement. This statement should provide the following information, where applicable:

- Accession codes, unique identifiers, or web links for publicly available datasets
- A description of any restrictions on data availability
- For clinical datasets or third party data, please ensure that the statement adheres to our policy

The raw Illumina sequencing data generated in this paper can be downloaded from the NCBI Sequence Read Archive under the BioProject Accession Number PRJNA820500. The extracted CDS sequences for each sample were deposited in the Mendeley Data Repository (Mendeley Data, V1, doi: 10.17632/mwyj4m963h.1).

Human research participants

Policy information about studies involving human research participants and Sex and Gender in Research.

Reporting on sex and gender	not applicable
Population characteristics	not applicable
Recruitment	not applicable
Ethics oversight	not applicable

Note that full information on the approval of the study protocol must also be provided in the manuscript.

Field-specific reporting

Please select the one below that is the best fit for your research. If you are not sure, read the appropriate sections before making your selection.

☐ Life sciences ☐ Behavioural & social sciences ☒ Ecological, evolutionary & environmental sciences

For a reference copy of the document with all sections, see nature.com/documents/nr-reporting-summary-flat.pdf

Ecological, evolutionary & environmental sciences study design

All studies must disclose on these points even when the disclosure is negative.

Study description	This study investigates the evolutionary history and diversification patterns of voles of Hengduan Mountains using phylogenomic data.
Research sample	Our study focus on evolutionary history of voles and the evolutionary relationship of these species. So we choose sample based on the taxonomy of species.
Sampling strategy	No sample-size calculation was performed. We choose sample based on the taxonomy of species.
Data collection	ShaoYing Liu, XuMing Wang, MingKun Tang and Yang Li carried out taxon sampling and collection by using traps from 1960 to 2017. Detailed information of collecting date is given in table S1.
Timing and spatial scale	Our taxon collection is from 1960 to 2017. The frequency and periodicity of sampling do not have regularity. The chance of meeting these species in the wild is very small and collecting these species is very difficult
Data exclusions	No data were excluded from the analyses.
Reproducibility	Most species contain only two samples to attempt to repeat the experiment.
Randomization	Our study do not need to random the samples. Our study focus on the evolutionary relationship of species. We just need to collect the sample of those species. We don't need to randomly sample those species.
Blinding	The investigators were blinded to group allocation duning data collection and analysis

Did the study involve field work? ☐ Yes ☒ No

Reporting for specific materials, systems and methods

We require information from authors about some types of materials, experimental systems and methods used in many studies. Here, indicate whether each material, system or method listed is relevant to your study. If you are not sure if a list item applies to your research, read the appropriate section before selecting a response.

Materials & experimental systems

n/a	Involved in the study
☒	☐ Antibodies
☒	☐ Eukaryotic cell lines
☒	☐ Palaeontology and archaeology
☐	☒ Animals and other organisms
☒	☐ Clinical data
☒	☐ Dual use research of concern

Methods

n/a	Involved in the study
☒	☐ ChIP-seq
☒	☐ Flow cytometry
☒	☐ MRI-based neuroimaging

Animals and other research organisms

Policy information about studies involving animals; ARRIVE guidelines recommended for reporting animal research, and Sex and Gender in Research

Laboratory animals	no laboratory animals
Wild animals	We use traps for sample capture in the field. If captive animals dies, we will keep the individual. If captive animals are alive, we take their fur and release them back into the wild.
Reporting on sex	we collected 73 male and 48 female vole and lemming specimens. Detailed information of sex on these samples is given in table S1. There is no Sex-based analysis, because sex is not considered in study design. Most species contain only two samples and cannot be analyzed at the species level.
Field-collected samples	Tissues were obtained from each specimen and preserved in 95% ethanol. Then the specimens were fixed in 10% buffered formalin, later transferred to 70% ethanol and stored at −20 °C for further use.
Ethics oversight	The related animal research content and methods involved in this study meet the ethical requirements of scientific research.

Note that full information on the approval of the study protocol must also be provided in the manuscript.
